# Aromatic Molecular Emitters in a Hexagonal Boron Nitride Stack

**DOI:** 10.1021/acs.nanolett.6c01870

**Published:** 2026-09-16

**Authors:** Tianyu Fang, Ricardo Gioia Alvarez, Babak Behjati, Moritz Scharfstädt, Max Masuhr, Bo Deng, Noah Henseler, Andrea Bergschneider, Stefan Linden, Christian Schäfer, Daqing Wang

**Affiliations:** † Institute of Applied Physics, 9374 University of Bonn, Wegelerstr. 8, 53115 Bonn, Germany; ‡ Physikalisches Institut, 9374 University of Bonn, Nussallee 12, 53115 Bonn, Germany; ¶ Institute of Applied Physics, Technical University of Vienna, Wiedner Hauptstr. 8−10, 1040 Vienna, Austria

**Keywords:** single-molecule spectroscopy, hexagonal boron nitride, polycyclic aromatic hydrocarbons, quantum emitters, van der Waals heterostructures, TDDFT

## Abstract

Single polycyclic aromatic hydrocarbon molecules embedded in organic matrices are excellent narrow line width quantum emitters. Extending this host–guest setting to van der Waals materials offers the opportunity to combine the preeminent properties of molecular emitters with access to the versatility of two-dimensional heterostructures. In this work, we incorporate perylene molecules into multilayered hexagonal boron nitride (hBN) stacks and observe gigahertz-narrow zero-phonon-line transitions at cryogenic temperatures. We unambiguously verify the origins of photon emission through vibronic spectra analysis. By combining hyperspectral localization measurements with quantum chemistry calculations, we examine the insertion mechanisms of perylene molecules in the stacks and conclude that pristine hBN layers tend to expel molecules from the stacks, while extended morphological defects, hydroxyl groups, and unpassivated boron and nitrogen atoms can assist to stabilize molecular bindings to hBN. Our work provides valuable insight for future work to deterministically integrate narrow line width molecular emitters into van der Waals devices.

## Graphical abstract

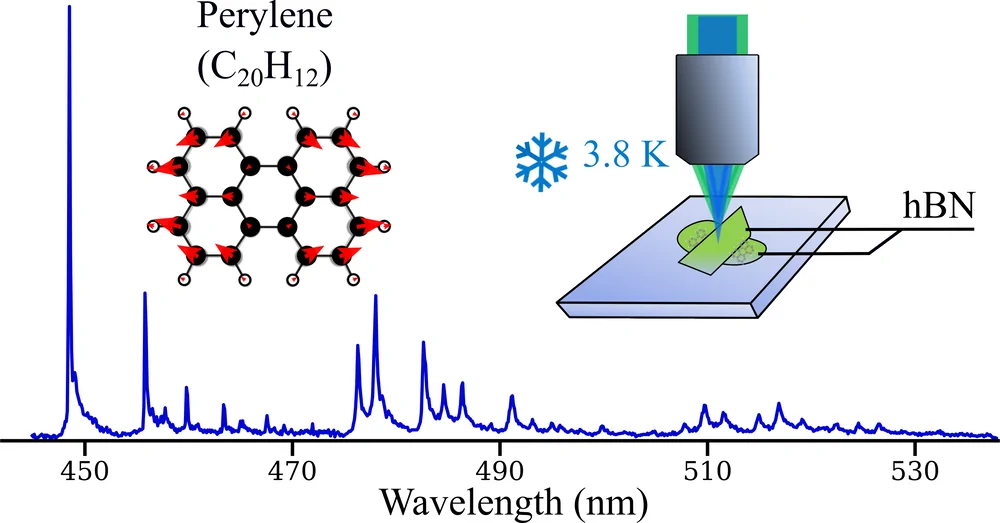

As a wide band gap two-dimensional material, hexagonal boron nitride (hBN) has recently gathered tremendous interest as a host material of single-photon emitters.^1,2^ hBN can be easily exfoliated down to a few layers, transferred onto a wide range of substrates, and incorporated into heterostructures and hybrid devices.^3^ A plethora of single-photon emitters in hBN has been discovered in the past decade.^4^ Some of them exhibit remarkable properties such as photon indistinguishability,^5^ room-temperature operation,^6^ and optically based spin initialization and readout.^7–9^ Yet, apart from a few species that could be reproducibly created or traced to identifiable atomic-scale structure,^7,10,11^ unambiguous assignment of the microscopic origin of most emitters remains an outstanding challenge.^12^

Concurrently, polycyclic aromatic hydrocarbon (PAH) molecules have established themselves as a class of well-understood and characterized single-photon emitters.^13,14^ They exhibit tunable, nearly Fourier-limited single-photon emission when embedded into organic matrices and cooled to liquid helium temperatures and are integrable into a wide range of photonic devices.^14^ The adaptability of PAH emitters into hBN has sparked recent attention.^15^ Dibenzoterrylene-doped anthracene nanocrystals have been integrated into heterostructures, where narrow line width and electrically tunable single-photon emissions have been demonstrated.^16^ The same architecture has been extended to enable the optical detection of trapped charges near a graphene transistor.^17^ Further recent studies have reported improvement of photostability of terrylene adsorbed on the surface of hBN^18^ and of TIPS-pentacene emitters placed on fused silica (SiO_2_) substrate and encapsulated by hBN,^19^ both at room temperature. At liquid helium temperature, narrow optical transitions down to 400 MHz have been measured on single terrylene molecules adsorbed on the hBN surface.^20,21^ On a different front, a certain family of hBN emitters with the emission energy at around 2 eV has been systematically verified to originate from organic molecules introduced by solvents and trapped between hBN and the SiO_2_ surface.^22^

These recent developments motivate the research question of whether PAH emitters can be directly inserted into hBN stacks without the need for nanoscale organic hosts and whether lifetime-limited molecular transitions can be detected. Answering these questions would open up possibilities for engineering hBN-based single-photon sources that are chemically tunable across a broad range of wavelengths and proximity-integrated molecular probes for studying local charge and strain dynamics. In seeking answers to these questions, we report a first, combined experimental and theoretical investigation of controlled incorporation of PAH molecules into hBN stacks.

Specifically, we chose perylene (C_20_H_12_), a small PAH molecule that emits in the blue, offset from the wavelength range of most common hBN emitters. In addition, perylene has been studied in various single-molecule experiments^23–26^ and features a comparatively noncomplex vibrational energy structure. We introduce perylene molecules to hBN through thermal evaporation. As shown in Figure 1, commercial hBN flakes purchased from 2D Semiconductors are first prepared on a silicon substrate (terminated with a SiO_2_ layer) through exfoliation. Perylene molecules are dissolved in chloroform at a mass ratio of 1:3100. A nickel–chromium alloy filament is immersed into the solution, dried, and then fixed on the electric feed-through of a vacuum flange, which is then attached to an inert gas chamber. A current passing through the filament produces ohmic heating and deposits perylene molecules onto the hBN flake (see SI section 1 for details about the evaporation process and sources of materials used). After evaporation, a second hBN flake, exfoliated on a clean substrate, is picked up by a stamp made of polydimethylsiloxane (PDMS) with a polycarbonate (PC) sacrificial layer and stacked onto the evaporated flake while the substrate is heated to 130 °C. For samples with high evaporation doses, the stack containing embedded molecules is picked up by the same stamp and transferred to a fresh substrate in order to isolate signals from molecules on the bare SiO_2_ surface. The stamp is detached by heating the substrate to 170 °C, leaving the stack and the PC layer on the substrate. After removing the PC layer with chloroform and cleaning with acetone, ethanol, and isopropanol, the substrate holding the stack is transferred into a closed-cycle helium cryostat and cooled to below 4 K for optical measurements. An additional baking step at up to 750 °C is applied to one sample to enable a controlled study of the effect of thermal annealing. A list of all samples and their processing steps is provided in SI section 1.

**Figure 1:**
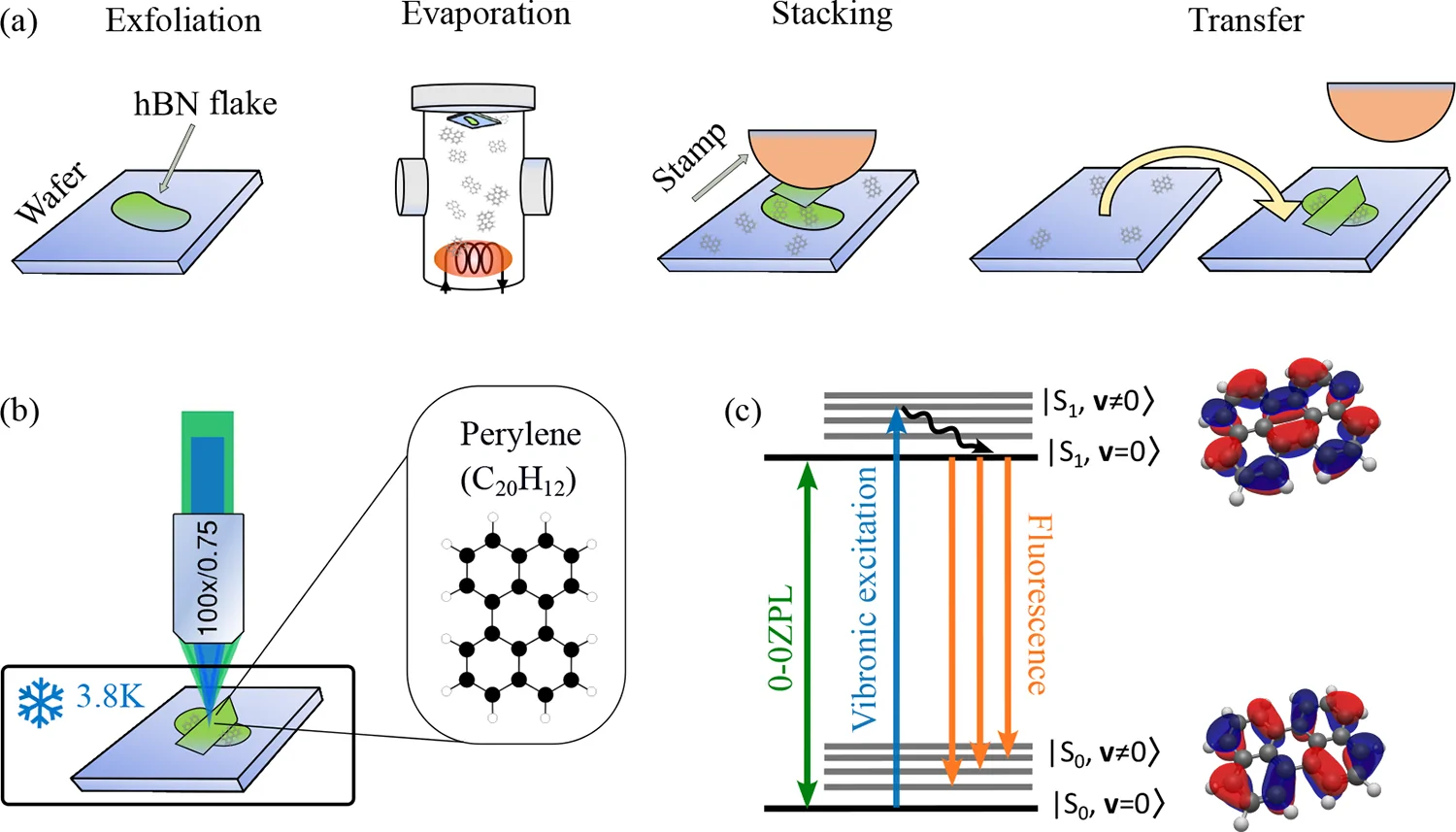
(a) Sample fabrication routine. An hBN flake is first prepared on a silicon wafer capped with a SiO_2_ layer. The substrate is then transferred into the evaporation chamber. After deposition, a second hBN flake is picked up by a polymer dome and stacked on the deposited hBN flake. The stack is picked up and transferred to a clean silicon substrate. (b) The sample is transferred to a closed-cycle cryostat, cooled to 3.8 K, and studied under an optical microscope. The inset shows the molecular structure of perylene. (c) A simplified energy level diagram of perylene. The two images on the right side display the π-orbitals involved in the |*S*_0_⟩ to |*S*_1_⟩ transition.

The photophysics of perylene molecules embedded in a solid is illustrated by the Jablonski diagram Figure 1(c). The electronic ground state |*S*_0_⟩ and the lowest singlet excited state |*S*_1_⟩ facilitate a strong optical transition, corresponding to the promotion of a π-electron to an excited orbital. Each of the two electronic states splits into a manifold |*S*_0,1_, **ν**⟩, where |**ν** = Π_*i*_ν^(*i*)^⟩ denotes the vector space supported by the vibrational modes of the molecule with *i* the mode index and ν^(*i*)^ = 0, 1, 2, ... the quantum number of occupation. The molecule can be optically excited from the vibronic ground state |*S*_0_, **ν** = **0**⟩ to a vibrational level of the excited state (blue arrow), from where it rapidly relaxes to |*S*_1_, **ν** = **0**⟩ (black curly arrow). The transition between |*S*_0_, **ν** = **0**⟩ and |*S*_1_, **ν** = **0**⟩ is called the 0–0 zero-phonon-line transition (0–0 ZPL, green arrow), and for simplicity, we refer to it as ZPL. Emissions to all other sublevels in the ground state manifold are allowed (orange arrows) with their relative strength described by the Franck–Condon factors. Measuring energy spacings among fluorescence emission lines thus allows reconstruction of the vibrational energy landscape of the ground-state manifold and enables unambiguous identification of the origin of optical emission.

We start with vibronic excitation of the sample with the laser frequency blue detuned with respect to the ZPL wavelengths of perylene measured in organic matrices.^23,24^ The stacked area is imaged on a scientific CMOS camera from which isolated fluorescent spots can be identified. Fluorescence light from each spot is spatially filtered in a confocal optical setup, coupled to a fiber and guided to a grating spectrometer. See SI section 1 for the camera images and SI section 2 for details of the optical setup. The signal shown in Figure 2(a) displays a representative emission spectrum. It features a series of dispersed sharp peaks, each accompanied by a sideband on the lower energy side. The sharp peaks resemble molecular emission features from |*S*_1_, **ν** = **0**⟩ to different |*S*_0_, **ν**⟩ sublevels, while the sideband associated with each peak is known as the phonon sideband induced by coupling to phonons in the host material.^14^ To validate that the emission is from perylene, we compare this spectrum with a reference measured from perylene molecules doped into the organic host anthracene, as shown in Figure 2(b). All identifiable vibronic peaks are in excellent agreement. The relative peak intensities and the shape of the phonon sidebands differ between the two cases, signifying different equilibrium geometries of perylene and different phonon densities of states in the two hosts. To further understand the microscopic origin of each peak, we calculate, with the ORCA code,^27^ the excited-state relaxation of perylene using time-dependent density-functional theory (B3LYP D4 def2-TZVPD)^28–31^ and estimate the vibrational modes of the hBN slabs using the universal machine-learning potential MACE-MP.^32^ The vibrational mode frequencies and the associated Franck–Condon factors are extracted from this combination and displayed by the blue vertical bars in the top panel of Figure 2(c). To obtain a clearer visual comparison, we convolve the peaks with the spectral response function of the spectrometer and an exponential function modeling the phonon sidebands (details about the model are given in section 4 of the SI). The resulting spectrum after a universal energy scaling by a factor of 1.109 is displayed by the blue curve. Shown below the theory curve are four additional perylene spectra measured from different samples. We find that almost all peaks can be explained by six modes and their overtones with up to three excitation quanta in each mode. The sketches in Figure 2(d) illustrate these six vibrational modes. Note that spectrum (iv) consists of doublets of each vibronic transition and thus likely originates from two perylene molecules in the same confocal spot. Figure 2(e) shows the second-order correlation measurement performed on one of the fluorescence spots. The antibunching behavior with *g* ^(2)^(0) around 0.5 is observed. Obtaining better counting statistics is, however, challenging because of the low quantum efficiency of photodetectors at 450 nm and the frequent photobleaching of the molecules.

**Figure 2:**
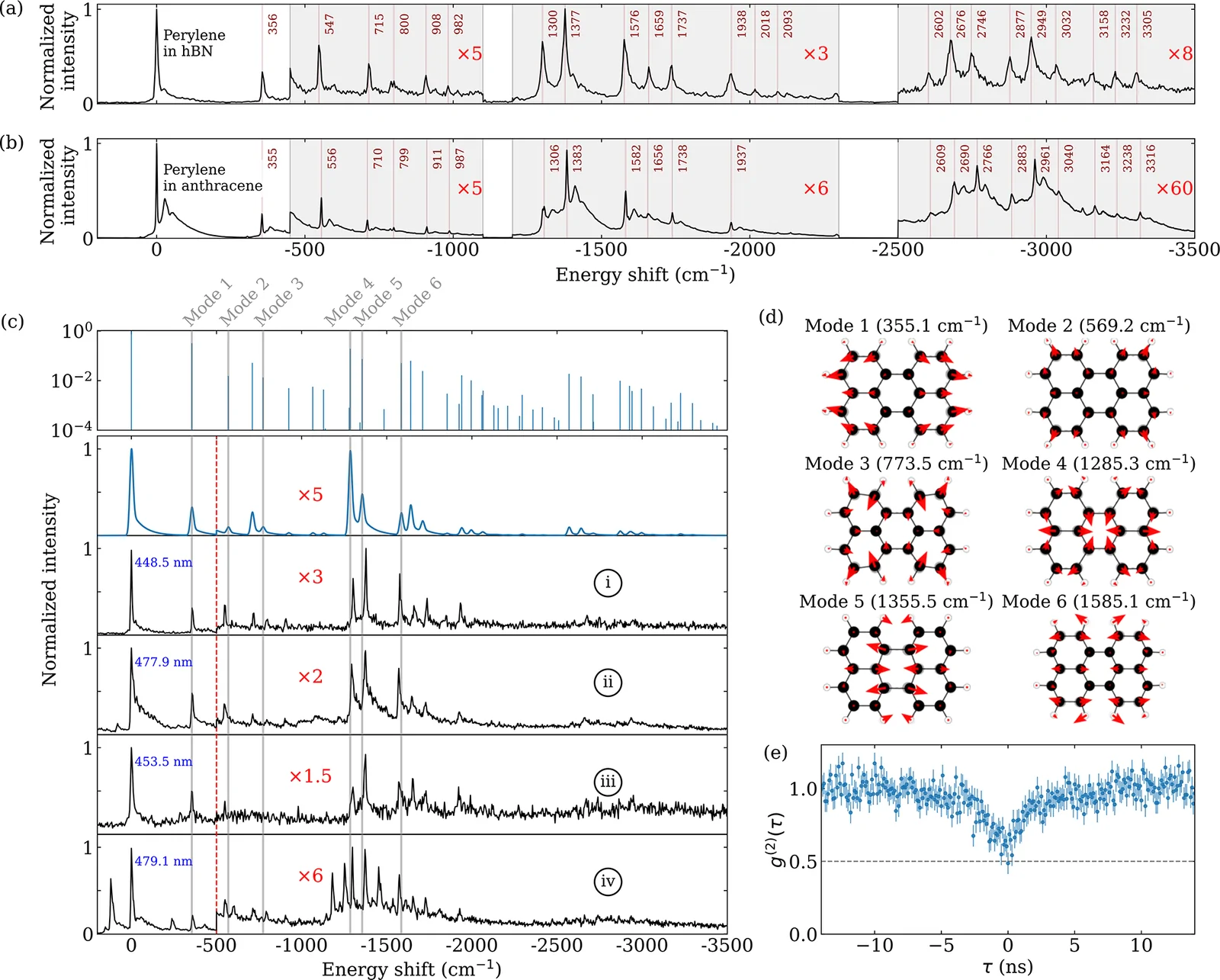
Fluorescence spectrum measured from a focal spot on the hBN stack (a) compared to the emission spectrum of perylene in anthracene (b). The horizontal axis denotes the relative energy shift to the ZPL, expressed in inverse centimeters. The vibronic bands are scaled differently in intensity for visual effect. (c) Comparison to theory. The blue vertical bars in the top panel present the vibrational modes of perylene in an hBN slab. The height of each bar represents the Franck–Condon factors derived from *ab initio* calculations. Only six modes and their overtones are plotted. The blue curve shows a modeled emission spectrum, which considers a broadening by the spectrometer and the phonon sideband. A universal energy scaling of 1.1 is performed to the theory data. The four spectra (i) to (iv) are measured at various locations on different samples. Spectrum (iv) features two sets of emission peaks, signifying that there are at least two molecules located in the optical focus. (d) Cartoons of the six vibrational modes and their calculated energy. The length of arrows illustrates the amplitude of the in-plane component of the acceleration experienced by each atom. (e) Normalized second-order correlation measurement of a focal spot on the encapsulated region.

Next, we extract the energies of these six modes from the measured spectra and compare them with the corresponding values obtained from our measurements of perylene in anthracene as well as with literature values reported for perylene in biphenyl.^23^ The results are displayed in Table 1. The extracted mode energies agree well with the reference values of perylene in anthracene and biphenyl with a root-mean-squared deviation of approximately 7.9 cm^–1^, corresponding to an averaged deviation below 1%. At the same time, modes 2 to 6 are systematically shifted to lower energies by about 7 to 9 cm^–1^, suggesting that the hBN environment induces a slight softening of these modes. These comparisons lead us to conclude that the observed emission features indeed originate from perylene molecules.

**Table 1:** Vibrational Mode Energies of Perylene in Anthracene and Biphenyl Compared to the Extracted Values from the Spectra in Figure 2(c) and the Calculated Values with MACE-MP Potential after a Global Scaling^a^

Mode Number	Biphenyl (cm^–1^)	Anthracene (cm^–1^)	M-i (cm^–1^)	M-ii (cm^–1^)	M-iii (cm^–1^)	M-iv.1 (cm^–1^)	M-iv.2 (cm^–1^)	DFT Calculation (cm^–1^)
1	358	355	356.4	357.4	355.9	358.5	360	355.1
2	557	556	549	546.5	549	550	553.5	569.2
3	808	799	796.5	795.5	–	791.4	–	773.5
4	1311	1306	1302.1	1293.5	1301.2	1298	1302	1285.3
5	1384	1383	1378	1374.2	1373.7	1374	1375	1355.5
6	1584	1582	1577	1570.8	1572.6	1574	1574.5	1585.1

a M-i to M-iv.2 denote the five sets of molecular emission features extracted from the four spectra.

Next, we study the spectral stability of the molecules, which can shed light on the stability of their local nanoscopic environment. Figure 3(a) displays the evolution of the emission spectrum obtained from a diffraction-limited fluorescence spot over a continuous measurement time of 220 s. This sample has not gone through thermal annealing. A careful analysis of the spectra (details in SI section 5) suggests that six separate perylene emission features are present. Figure 3(b) shows the trajectories of the ZPL transition (opaque lines) of the molecules and their associated vibronic lines (semitransparent lines). The three frequency traces labeled 1–3 on the left side of the plot all exhibit strong spectral diffusion. The vibronic emission lines shift synchronously with their respective ZPLs. This suggests that the spectral shifts are due to electronic coupling to their environment instead of changes involving mechanical distortions, such as hopping between binding sites. The latter would lead to pronounced modifications to the vibrational mode energies and Franck–Condon factors. Further observations show that molecules 2 and 3 exhibit very similar diffusion patterns that differ from that of molecule 1, indicating that molecules 2 and 3 experience similar local environments that are different from the environment of molecule 1. Molecule 4 has remained mostly stable. Molecules 5 and 6 show interesting correlations in their emission intensity. Toward the end of the measurement sequence, molecule 6 suffered from photobleaching. At the same time, an increase in intensity appears in the ZPL and vibronic lines of molecule 5. This observation can be explained by Förster resonance energy transfer (FRET). Molecule 5, having higher energy, functions as a donor and has certain probability to transfer its excitation to the energetically lower-lying molecule 6, the acceptor. Photobleaching of organic fluorophores is associated with the breaking of the π-delocalization via a chemical reaction, which leads to a significant increase in the absorption energy gap.^33^ When the acceptor molecule is bleached, its excited-state energy shifts above that of the donor. The resonance condition of FRET is no longer fulfilled, and the fluorescence quantum yield of molecule 5 increases, leading to its increase in brightness. Since the efficiency of FRET is inversely proportional to the sixth power of the intermolecular distance,^34^ our observation that molecule 5 becomes approximately a factor of 2 brighter after molecule 6 has undergone photobleaching implies a donor–acceptor separation close to the Förster radius, which should amount to several nanometers.

**Figure 3:**
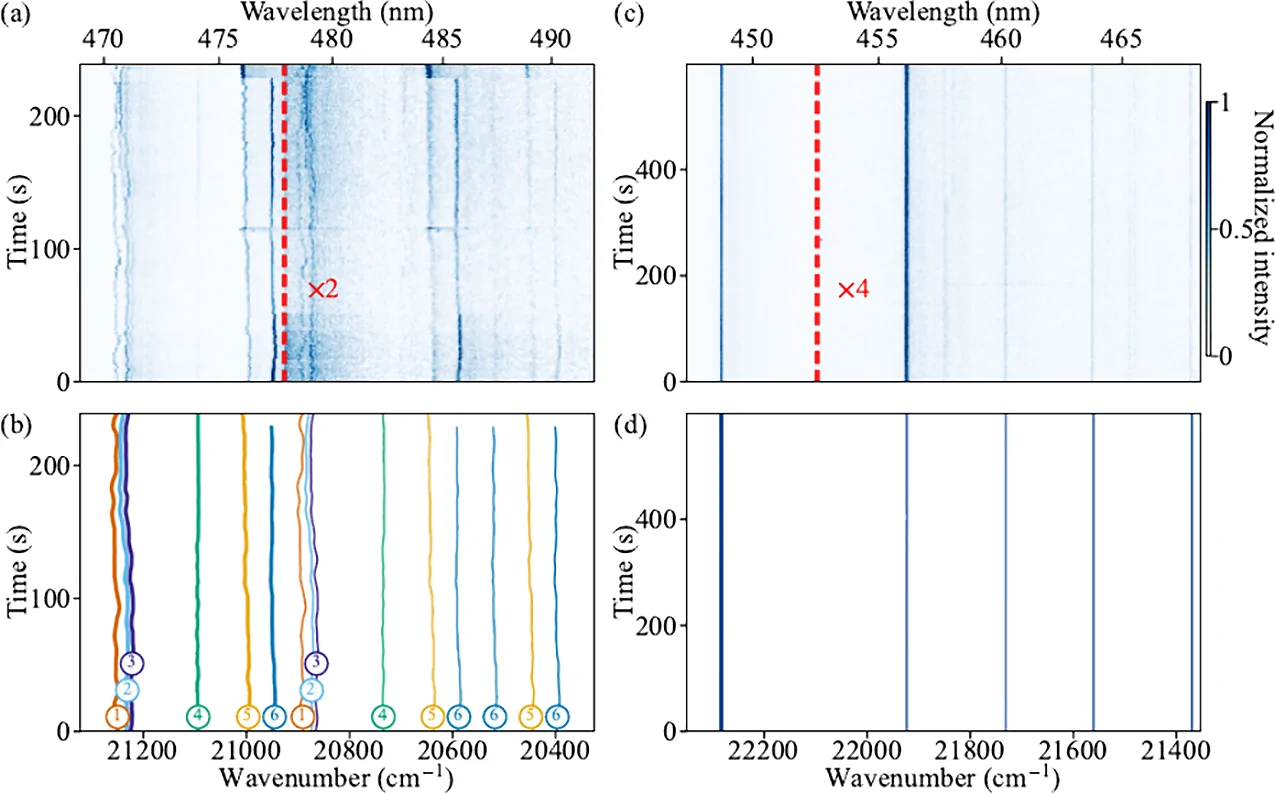
(a) Emission spectrum measured on a laser focal spot on a non-baked sample over 200 s. (b) Extracted frequency traces of 6 molecules. Each molecule is represented by one color. The opaque lines denote the ZPL transition, and the semitransparent lines are the vibronic transitions. (c) An emission spectrum measured on a sample after baking. (d) Extracted time traces of the ZPL and vibronic lines for the data in (c).

The observation of photobleaching suggests the presence of reactive species in the local intermediate of the molecules. During sample preparation, water and oxygen molecules can be trapped within the sandwich, or adsorbed at defect sites, and can thus act as reactants leading to oxidation of the acceptor molecule. In addition, the rotation or diffusion of these small molecules may contribute to spectral diffusion of the perylene. To test this hypothesis, we perform a control experiment by baking one of the hBN stacks at 750 °C in air for a duration of 4 h. At this temperature, we expect that small molecules like water or oxygen can still diffuse through and escape the stack. Shown in Figure 3(c) is a perylene emission spectrum measured on the baked sample, and Figure 3(d) illustrates the respective spectral traces of the ZPL and vibronic lines. Here, we observe no significant intensity change and spectral drift over the duration of 10 min. This observation supports the conclusion that removing small adsorbate molecules reduces environmental fluctuations and thereby creates a more stable local environment.

We next perform resonant excitation to probe the linewidth of the ZPL transition. To do so, the excitation laser is tuned to 476 nm. The fluorescence signal from |*S*_1_, **ν** = **0**⟩ to different |*S*_0_, **ν** ≠ **0**⟩ transitions is separated from the excitation light with a set of long-pass interference filters and measured by an avalanche photodetector, while the laser frequency is scanned across the ZPL transition of a molecule. Figure 4(a) shows the measured fluorescence excitation spectra of a molecule. In this set of measurements, the excitation power is increased stepwise from 0.8 nW to 1.14 μW (measured before the cryostat window). Figure 4(b) presents the extracted excitation spectra at different powers. A linewidth of around 2.8 GHz is observed without noticeable power broadening. As the laser intensity increases to 1.14 μW, fluorescence counts of up to 80,000 per second can be detected. However, a spectral jump occurs at this laser power, which signifies a photoinduced change in the local environment of the molecule. The abrupt on–off change in the fluorescence intensity evidences that the feature originates from a single molecule. The observed linewidth is considerably broader than single perylene molecules measured in biphenyl (140 MHz, ref 23) and *n*-nonane (28 MHz, ref 35), implying a stronger pure dephasing experienced by the molecule in hBN. The linewidth dominated by pure dephasing also explains the absence of noticeable power broadening in the linewidths measured in Figure 4(b).

**Figure 4:**
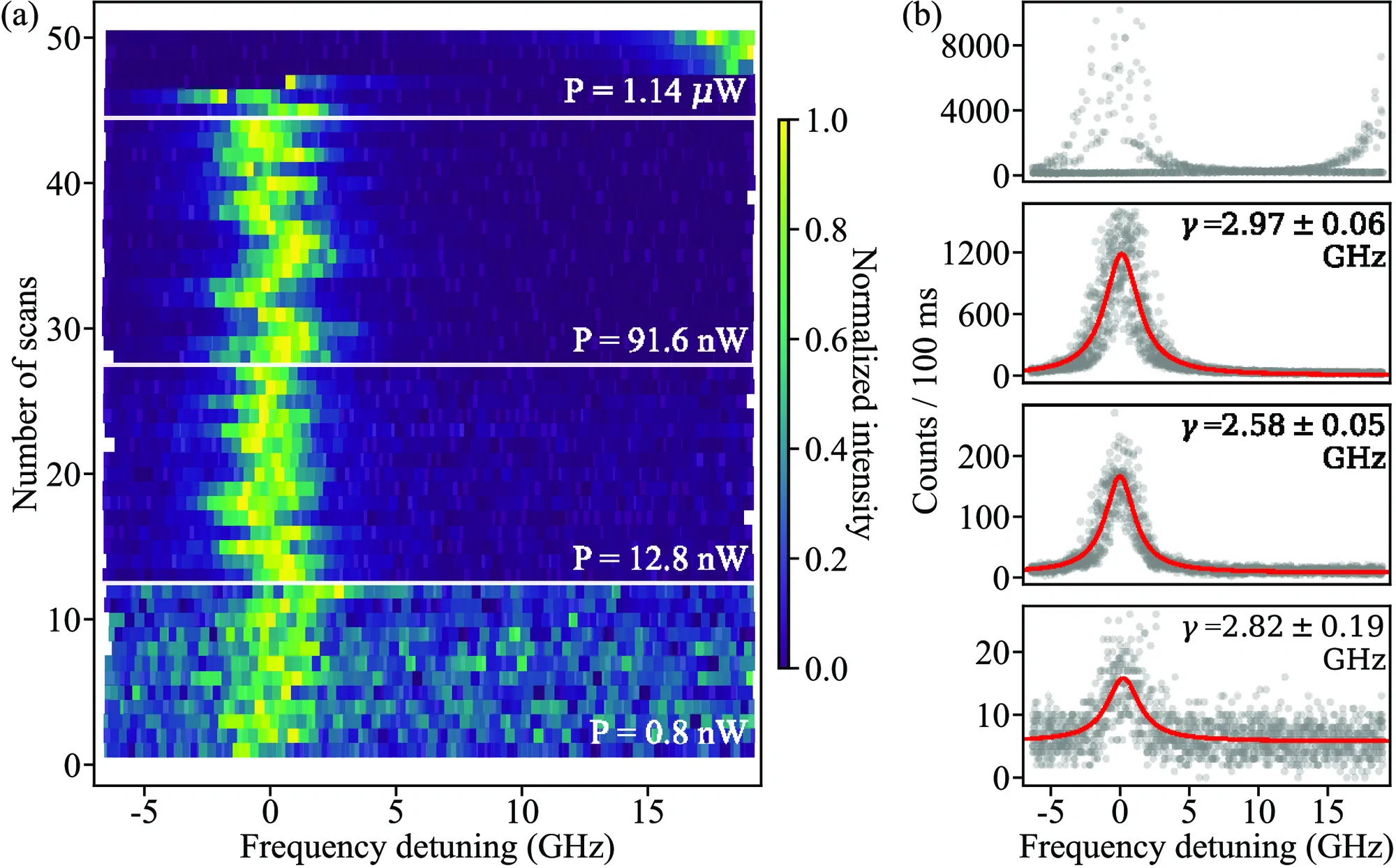
Fluorescence excitation spectra of the ZPL transition of a single perylene molecule. (a) The ZPL excitation traces measured with gradually increased laser intensities. The duration of a single frequency sweep was 12 s. The color scale is normalized in each scan. Spectral jumps occur at the highest excitation power. (b) Excitation spectra at each laser power. The gray dots show the accumulated data from (a) without postaligning the center resonance frequency of each individual scan. The red lines denote fits to a Lorentzian function. The extracted full-width at half-maximum linewidth γ is given in the legend.

An important open question is about the insertion geometries of perylene molecules in the hBN stack. To gain experimental insight, we perform spatially resolved emission spectrum measurements on the sample. To do so, the laser focus is raster scanned across the sample and an emission spectrum is acquired at each position. The results are analyzed in correlation with topographic measurements using an atomic force microscope (AFM). Figure 5(a),(e) shows the white-light microscope images of two samples, where the boundaries of the bottom and top hBN flakes are marked with orange and blue lines, respectively. The area in the white square is studied. The results from the AFM measurements are shown in Figure 5(b),(f), respectively. The bright stripes in both images are bulges formed during exfoliation and stacking. The labels (i), (ii), and (iii) denote areas of the hBN-stack, SiO_2_ surface covered by the top flake, and bare SiO_2_ surface, respectively. Figure 5(c),(g) displays the fluorescence intensity maps obtained by integrating the spectrum measured at each position. One can readily identify that areas stacked between flat hBN flakes show weaker fluorescence intensity compared to areas with bulges, flake edges, and substrate areas that are covered by the top hBN layer. To specifically trace perylene signals, we use the vibronic fingerprints in the range of 1200 cm^–1^ to 1800 cm^–1^ of perylene to perform an intensity-normalized cross-correlation analysis with the hyperspectral data set (see SI section 7 for details). The resulting correlation maps are displayed in Figure 5(d),(h). In the areas stacked between flat hBN layers, individual scattered spots are identifiable. The areas with major morphological irregularities, i.e., flake edges and bulges, appear to be much brighter than the stacked area. These regions can present complex nanoscopic environments, including high densities of hBN defects as well as residual organic compounds from the stacking process. The favorable binding of perylene molecules in these regions is likely not entirely hBN-related. The areas on the SiO_2_ surface and covered by the top hBN layer [area (ii)] also feature a strong intensity in the correlation map, which suggests that the SiO_2_ surface offers more favorable bindings for molecules compared to pristine hBN. It is to note that the stacks in (a) and (e) were both transferred to a fresh substrate before the optical measurements. The observation of perylene molecules in these areas indicates that these molecules were picked up by the top flake from the initial substrate during the detaching process.

**Figure 5:**
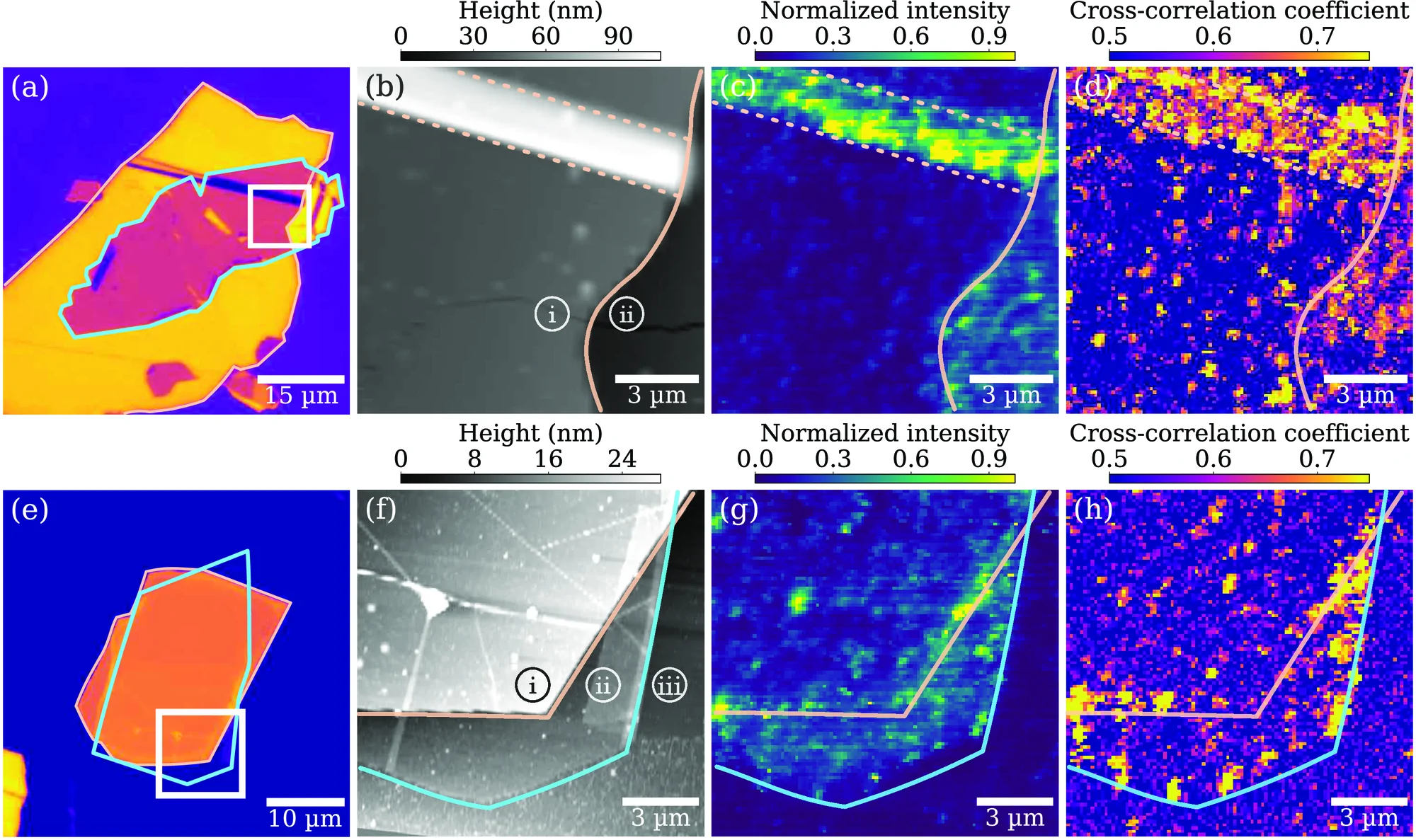
Spatial distribution of perylene emissions analyzed through hyperspectral measurements on two samples. (a), (e) Optical microscope images of the two samples. Orange and blue contours illustrate the boundaries of the lower and upper hBN flakes, respectively. (b), (f) Atomic force microscope measurements. The top and bottom flakes in (b) have a thickness of 21 and 31 nm, respectively. The sample in (f) has a 22 nm-thick bottom flake and a 2 nm-thick top flake. (c), (g) Integrated fluorescence intensity distributions. (d), (h) Normalized correlation function with respect to the emission spectrum of perylene.

Through hyperspectral analysis, we also extract the distribution of ZPL wavelengths (see SI section 7, Figure S21). Across all samples prepared, we identify perylene ZPLs in the range of 440 to 500 nm. The distribution can be primarily categorized into a main peak between 445 and 470 nm and a broader, more sparsely distributed group between 470 and 500 nm. We further examined whether the observed ZPL heterogeneity is correlated with the morphology of the hBN flakes by comparing perylene molecules located in flat stacked regions and those found in the morphologically irregular regions. The results are displayed in Figure 6. The two groups exhibit broadly similar ZPL distribution trends with no clear systematic shift or additional broadening. For comparison, the ZPL wavelengths of perylene molecules embedded in organic matrices lie in the range of 444 to 454 nm.^36^ The broader ZPL distribution observed in the perylene-hBN system therefore indicates substantially more heterogeneous insertion configurations of perylene. The significant red shift of the ZPLs in the range of 475 to 500 nm could be due to defect-related binding. Coupling to defect sites in hBN may differentially stabilize the electronic ground and excited states of perylene, leading to a decrease in the optical transition energy. A red shift of the ZPL has also been observed for terrylene molecules adsorbed on hBN surfaces.^20^

**Figure 6:**
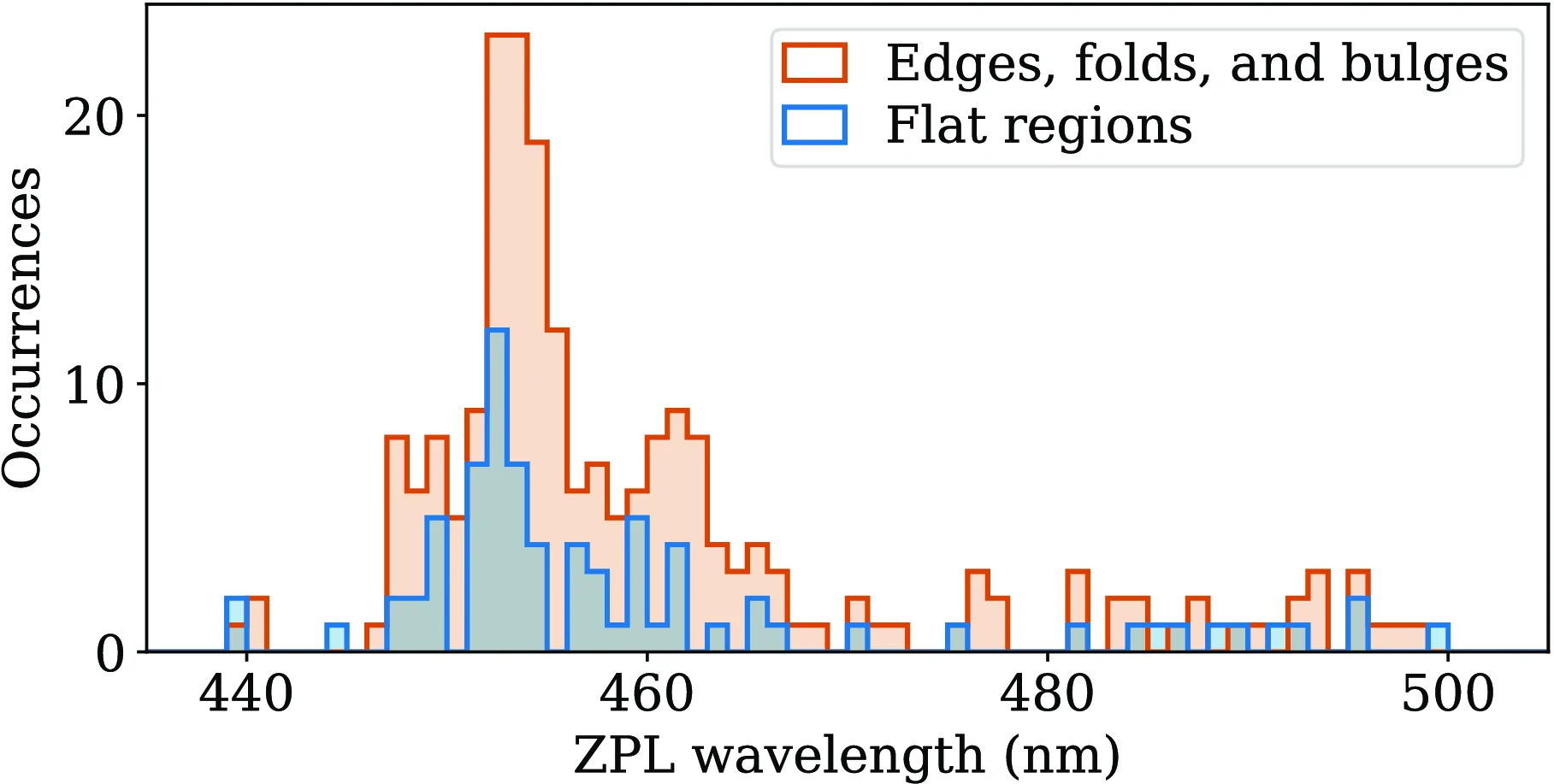
ZPL wavelength distributions of perylene molecules located in flat stacked hBN regions and in morphologically irregular regions, including flake edges, folds, and bulges.

We seek to gain a microscopic understanding of possible binding geometries by numerically relaxing the combined perylene-hBN structure. While the influence from residual organic compounds cannot be excluded, they are unknown in chemical composition and structure and are thus not the focus of our simulations. We model the perylene-hBN system using the ASE software package^37^ in combination with the foundation machine-learning interatomic potential MACE-MP (medium size)^32^ and explicitly account for dispersive interactions. We create various possible defects and relax all cell parameters and nuclear positions for 50,000 steps using the FIRE optimization algorithm. The stability on insertion is benchmarked by calculating the formation energy (or binding energy of perylene) *E*_f_ = *E*_Mol+hBN_ – (*E*_Mol_ + *E*_hBN_). The results are presented in Table 2, and all corresponding structures are visualized in section 3 of the SI. As a benchmark of the computational approach, we calculated the adsorption energies of perylene and terrylene on a pristine monolayer hBN surface. We obtain formation energies of *E*_f_ = −1.72 eV and −2.32 eV for perylene and terrylene, respectively, in good agreement with the adsorption energy of −2.44 eV reported for terrylene on hBN by de Haas et al.^21^ The stronger adsorption of terrylene compared to perylene is furthermore consistent with previous numerical studies showing an increase in the adsorption energy of PAH molecules with increasing numbers of aromatic rings.^38^ We found that sandwiching perylene between two pristine monolayers of hBN always leads to a positive formation energy in the order of 5.5 eV [E1 in Table 2 and Figure 7(a)], suggesting that it is energetically expensive to sandwich perylene molecules between pristine layers. Single point defects in the hBN lattice, including B- and N-vacancies and oxygen- or carbon substitutions (E2–E5), lead to only marginal modifications to the formation energy. Extending the bilayer to multilayer hBNs increases the formation energy further (E6–E7). To estimate the energetic barrier for lateral motion of perylene within the stack, we perform a further set of simulations, where the lateral position of the perylene molecule is fixed along a path across a unit cell of hBN, while the hBN stack is allowed to relax. The results give an estimated migration barrier of *E*_b_ = 54.6 meV. This value is larger than the thermal energy at room temperature, which amounts to 25.9 meV at 300 K, but not by orders of magnitude. The corresponding Arrhenius activation factor is exp­(−*E*_b_/*k*_B_ *T*) ≈ 0.12, suggesting that lateral motion is thermally accessible. During stacking and delamination, the sample is further heated and the activation factor increases to 0.24. The zipper-like closure of the interface can mechanically assist the displacement or expulsion of weakly bound molecules. These findings are in accordance with previous studies indicating that the stacking process expels hydrocarbon molecules from hBN surfaces.^39,40^

**Figure 7:**
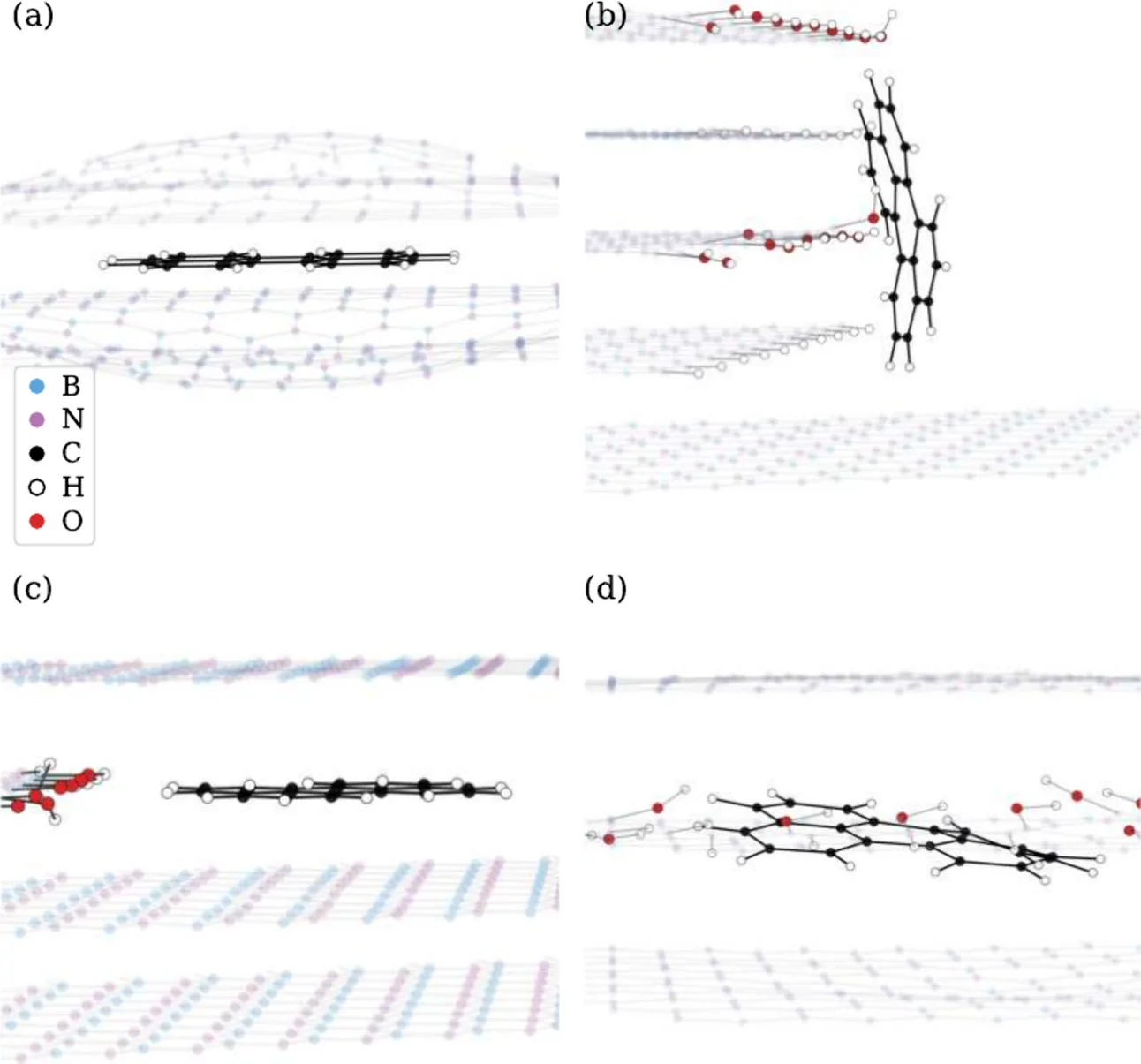
Geometry of the relaxed molecule-hBN structure. (a) E1, perylene sandwiched in bilayer hBN, (b) E10, multilayer hBN edge, (c) E14, embedded step terrace, and (d) E16, extended vacancy. The structures in (b), (c) and (d) have −H terminated nitrogen and −OH terminated boron atoms.

**Table 2:** Formation Energies of Perylene Molecules Inserted in Pristine and Defective hBN Structures^a^

ID	Structure	Termination/Defect	Formation Energy (eV)
E1	Bilayer stack (1-Pr-1)	pristine	5.548
E2	Bilayer stack (1-Pr-1)	B vacancy	5.151
E3	Bilayer stack (1-Pr-1)	N vacancy	5.764
E4	Bilayer stack (1-Pr-1)	substitute N by O	5.508
E5	Bilayer stack (1-Pr-1)	substitute B by C	5.528
E6	Multilayer stack (4-Pr-4)	pristine	8.215
E7	Multilayer stack (4-Pr-4)	substitute N by O	8.143
E8	Multistep edge (4-Pr-4)	no termination	–
E9	Multistep edge (4-Pr-4)	no termination	–
E10	Multistep edge (4-Pr-4)	OH termination	–1.114
E11	Embedded step (3-Pr-1)	no termination	–3.645
E12	Embedded step (3-Pr-1)	H termination	–0.770
E13	Embedded step (3-Pr-2)	H termination	–0.738
E14	Embedded step (3-Pr-1)	OH termination	–2.601
E15	Extended vacancy (5-Pr-3)	no termination	2.132
E16	Extended vacancy (5-Pr-3)	OH/H termination	–3.940

a The notation *m*-Pr-*n* denotes a structure with perylene molecules binding to *m* layers of bottom hBN and encapsulated with *n* layers of top hBN. Formation energies for E8 and E9 could not be precisely captured due to random formations of interlayer B–N bonds at flake edges.

We further explored the influence of extended defects, including multistep edges [E8–E10 and Figure 7(b)], embedded monostep terraces [E11–E14 and Figure 7(c)], and extended vacancies [E15–E16 and Figure 7(d)]. Negative formation energies can be obtained in all these structures. A further examination of the results shows that nonterminated boron atoms tend to covalently bind to carbon atoms in the molecule, and nonterminated nitrogen atoms stabilize the molecule through short-range interactions with the peripheral C–H groups of perylene. This leads to rather large negative formation energies (E8, E9, E11). The formation energies for E8 and E9 could not be precisely captured by our numerical routine due to the random formation of interlayer covalent bonds of boron and nitrogen atoms at the edge of the flake (see SI section 3, Figure S10). Physical bindings are found when the dangling boron and nitrogen atoms are terminated. Terminating boron atoms by hydroxyl groups (E10, E14, E16) leads to considerably stronger binding than complete hydrogen terminations (E12, E13), suggesting that the −OH−π interaction^41,42^ potentially stabilizes the insertion of perylene. In our experiments, exfoliation and stacking are performed under ambient conditions. Hydroxyl-terminated defects are expected to form more readily through reactions with water molecules than unpassivated radical sites or hydrogen-terminated defects. Direct covalent binding to the molecule, in particular B–C bond formation, would partially disrupt the π-delocalization of perylene. This would be expected to strongly modify both the optical transition energy and the vibrational spectrum and is therefore unlikely to be captured by our hyperspectral analysis.

The correlation map presented in Figure 5, in which perylene signals are primarily found at the edges and extended structural defects, is consistent with the calculation results. In addition, the areas sandwiched between hBN and SiO_2_, which are rich with perylene signals, are also reflected by the simulation results, as the SiO_2_ surface is known to be rich with hydroxyl terminations under exposure to humidity under ambient conditions.^43^ The sparse distribution of perylene molecules in the stack area should be most likely due to extended vacancies and defects on the upper or lower hBN flakes.

Overall, in this work, we performed a combined experimental and theoretical investigation of perylene molecules incorporated into hBN stacks. The choice of perylene, one of the smallest and well-characterized fluorophores in the PAH family, allows us to unambiguously identify their vibronic emission fingerprints. The main conclusions of this work are two-fold. First, cryogenic optical spectroscopy shows that perylene molecules can be unambiguously identified, and a narrow zero-phonon-line optical transition down to 2.8 GHz can be measured. Second, experimental results and theoretical calculations suggest that stable binding of molecules in hBN should be primarily defect-enhanced. The results of hyperspectral analysis show that perylene insertion locations are strongly correlated with morphological irregularities in the structure, such as flake edges and wrinkles. These findings are consistent with the self-cleaning mechanism proposed for stamp-based van der Waals assembly in which weakly bound hydrocarbon adsorbates are expelled from pristine interfaces.^39,40^ In this context, defects in hBN can act as local trapping sites that stabilize molecules against expulsion.^44^

Our work can be viewed as a first step toward deterministic integration of molecular emitters into hBN. A line of future work is to be performed to make the system attractive for coherent quantum optical experiments. First, although narrow ZPL transition at the gigahertz level is observed, the holy grail of achieving a lifetime-limited line width, which is necessary for implementing coherent quantum protocols, still requires further investigation. To improve in this aspect, microscopic understanding of pure dephasing and spectral diffusion mechanisms is required. Here, insights may be gained from works on native hBN defects, where charge noise was found to be a main leading mechanism.^45^ Future effort could incorporate electric gating structures to regulate the local electric field environment at the molecule. Second, the density of isolated single molecules is much lower than in typical organic host matrices. The two-dimensional geometry provides fewer possible insertion sites than a three-dimensional crystal, and the stacking process likely expels most perylene molecules from pristine regions of the interface. Specifically, when comparing our observation with the established organic host–guest system of dibenzoterrylene embedded in anthracene, which can show on the order of 100 isolated emitters per cubic micrometer,^46^ we find the density of isolated emitters in perylene/hBN to be substantially lower with an average of approximately 50 observable zero-phonon lines within a 10 × 10 μm^2^ area. In addition, the photostability of perylene/hBN is currently limited to several minutes under continuous excitation. In contrast, dibenzoterrylene molecules embedded in anthracene are well-known for their exceptional photostability, routinely allowing repeated measurements without observable photobleaching at cryogenic temperatures. The reduced stability of perylene in hBN is likely related to the less chemically inert hBN interface and the higher excitation energy of perylene, which increases the probability of photochemical reactions. Moreover, perylene is relatively volatile among PAHs and can exhibit high surface mobility, making systematic control of the molecular density challenging. In future experiments, larger molecules with lower surface mobility, such as PTCDA or TIPS-pentacene, could be used. Improved dose control may also be achieved by physical vapor deposition under vacuum.^47^ In addition, controlled introduction of molecules may be achieved by controlling the location and density of extended defects, which can be achieved through, e.g., surface self-assembly^48^ or nanoindentation^49^ prepared hBN surfaces. Finally, the good agreement between theory and experiment on perylene suggests that predictive theoretical schemes in combination with global optimization techniques hold potential to uncover the vast chemical space of molecular emitters for predictable integration into two-dimensional materials.^50^

## Supplementary Material



## Data Availability

The data supporting this study are openly available in the Zenodo repository (https://doi.org/10.5281/zenodo.19665755).

## Notes

Sample preparation details; experimental setup; numerical computation methods, perylene–hBN insertion geometries, formation energies, and molecular diffusion barriers; Franck–Condon simulations, phonon-sideband modeling, and temperature-dependent emission spectra; spectral diffusion analysis, sample annealing, and hyperspectral imaging analysis (PDF)
