# Supplementary material for: Aromatic Molecular Emitters in a Hexagonal Boron Nitride Stack
Source: Nano Lett. 2026 Sep 16;26(38):12628–37. doi: 10.1021/acs.nanolett.6c01870 (PMC13632586; doi:10.1021/acs.nanolett.6c01870)
Supplement: Supplementary file 1 [file nl6c01870_si_001.pdf]

Supplementary Information for Aromatic molecular emitters in a hexagonal boron nitride stack

Tianyu Fang,† Ricardo Gioia Alvarez,† Babak Behjati,† Moritz Scharfstädt,‡ Max Masuhr,† Bo Deng,† Noah Henseler,† Andrea Bergschneider,‡ Stefan Linden,‡ Christian Schäfer,¶ and Daqing Wang*,†

†*Institute of Applied Physics, University of Bonn, Wegelerstr. 8, Bonn, Germany*

‡*Physikalisches Institut, University of Bonn, Nussallee 12, Bonn, Germany*

¶*Institute of Applied Physics, Technical University of Vienna, Wiedner Hauptstr. 8-10, Vienna, Austria*

E-mail: daqing.wang@uni-bonn.de

Contents

1 Sample preparation **1**

1.1 Evaporation of perylene molecules 1

1.2 Fabrication of hBN stacks 3

1.3 Fluorescence images 7

1.4 Perylene in anthracene sample fabrication 8

2 Experimental setup **9**

3 Numerical calculations **11**

3.1 Visualization of insertion geometries 11

3.2 Diffusion barrier of perylene in an hBN stack 17

4 Emission spectra **18**

4.1 Calculation of Franck-Condon factors 18

4.2 Phonon broadening model 20

4.3 Temperature-dependent emission spectra 21

5 Spectral diffusion data analysis **23**

6 Sample Annealing **27**

7 Hyperspectral imaging **28**

7.1 LNCC matching 30

7.2 Peak-spacing matching 31

1 Sample preparation

1.1 Evaporation of perylene molecules

The evaporation of perylene molecules is based on ohmic heating of a resistive nickel-chromium filament inside an evaporation chamber, as illustrated in Fig. **S1**. The filament is dipped into a solution of perylene (Sigma Aldrich, product number: 394475, purity $\geq 99.5\%$) in chloroform with a mass ratio of 1 : 3100 for 1 minute. Before each evaporation run, the chamber is repeatedly evacuated and flushed with nitrogen gas.

Afterwards, the chamber is either put under a low vacuum of around 100 mbar, or under overpressure in nitrogen. For the latter case, the exact overpressure was not recorded. In this way, we vary the mean free path of perylene molecules in order to control the deposition yield on the sample. The evaporation times range from a few seconds to one minute for a

low vacuum of around 100 mbar, and many minutes for an overpressure in the chamber (see table **S1** for parameters for each sample). For overpressure environments, currents between 600 mA and 1 A are applied, while for a low vacuum, we use currents between 50 mA and 1 A. The resistances of the different filaments used range from 2 Ω to 8 Ω.

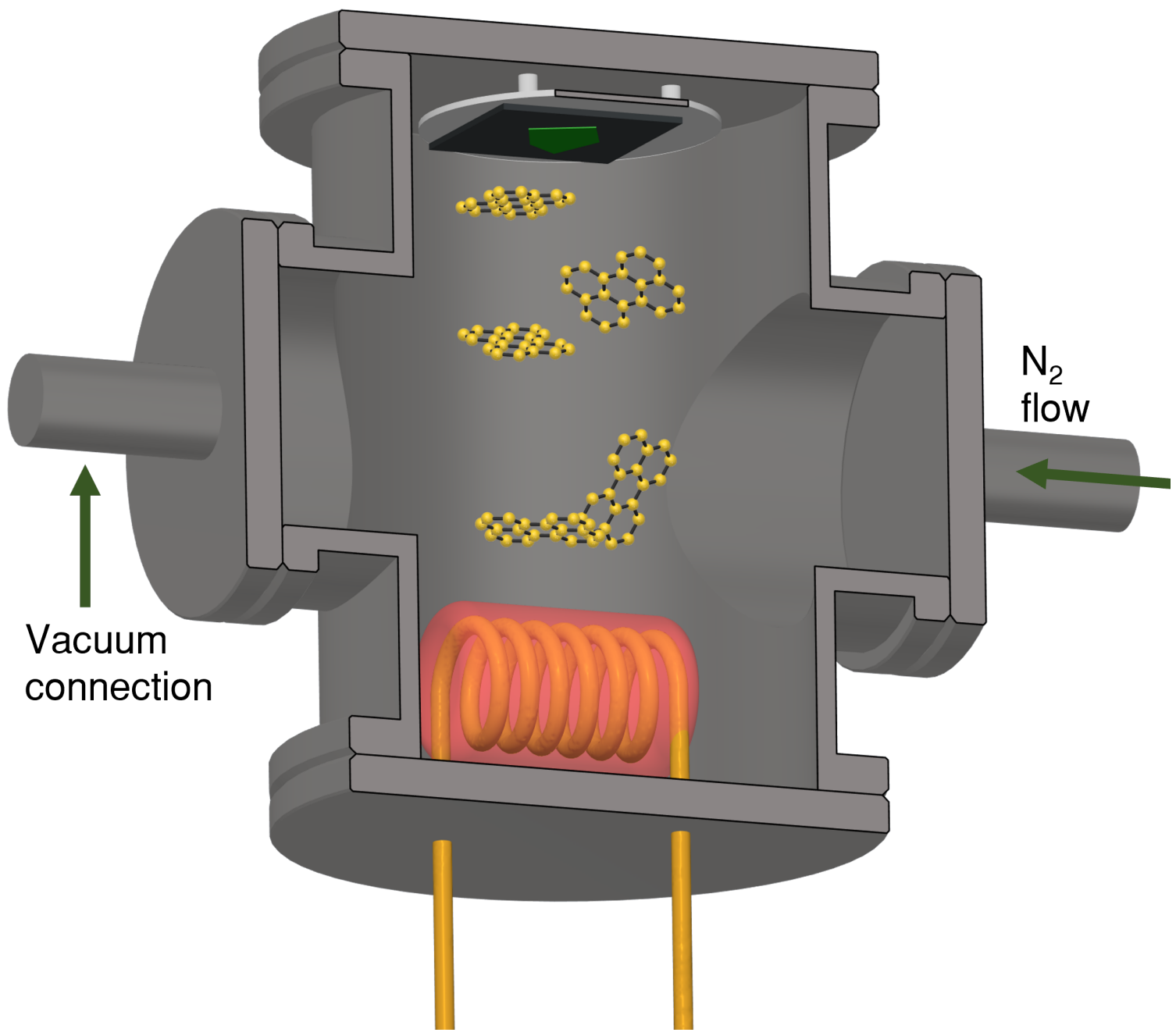

Figure S1: Sketch of the evaporation process inside the chamber. In the top part of the chamber, a substrate holding hBN flakes is mounted onto a chip holder. The vacuum and nitrogen gas connections are equipped on the two sides. The filament at the bottom is being heated, where molecules are evaporated.

In Tab. **S1**, different sample preparation parameters for the corresponding samples are shown. Images of an example hBN flake before and after evaporation are shown in Fig. **S2**. Several optical images of parts of the filaments before and after an evaporation run are presented in Fig. **S3**. For Figs. **S2** and **S3**, the observation was done with a custom-built room temperature microscope that utilizes a LED as an excitation source with peak emission at 425 nm. For fluorecence imaging, 442 nm long-pass and 450 nm long-pass filters were utilized in the emission path.

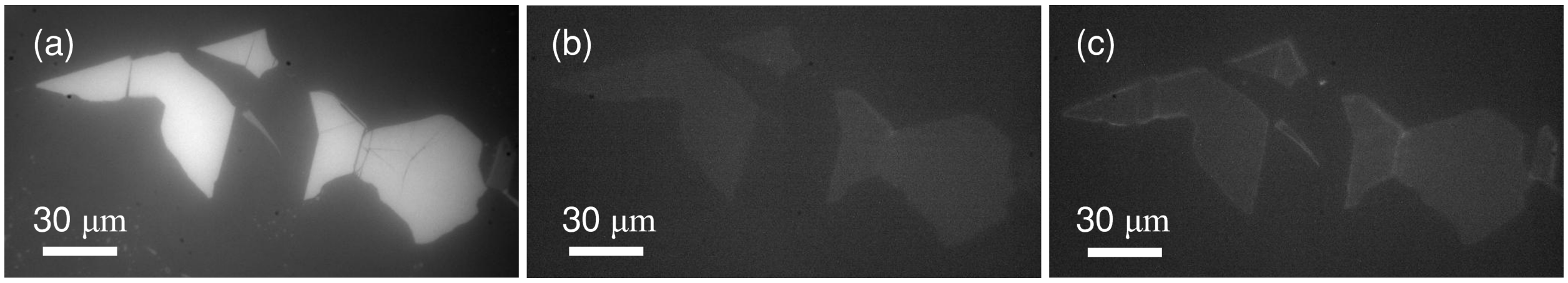

Figure S2: (a) White-light and (b) fluorescence image of exemplary hBN flakes before evaporation. (c) Fluorescence image of the same flakes directly after an evaporation run. An increase in fluorescence can be observed.

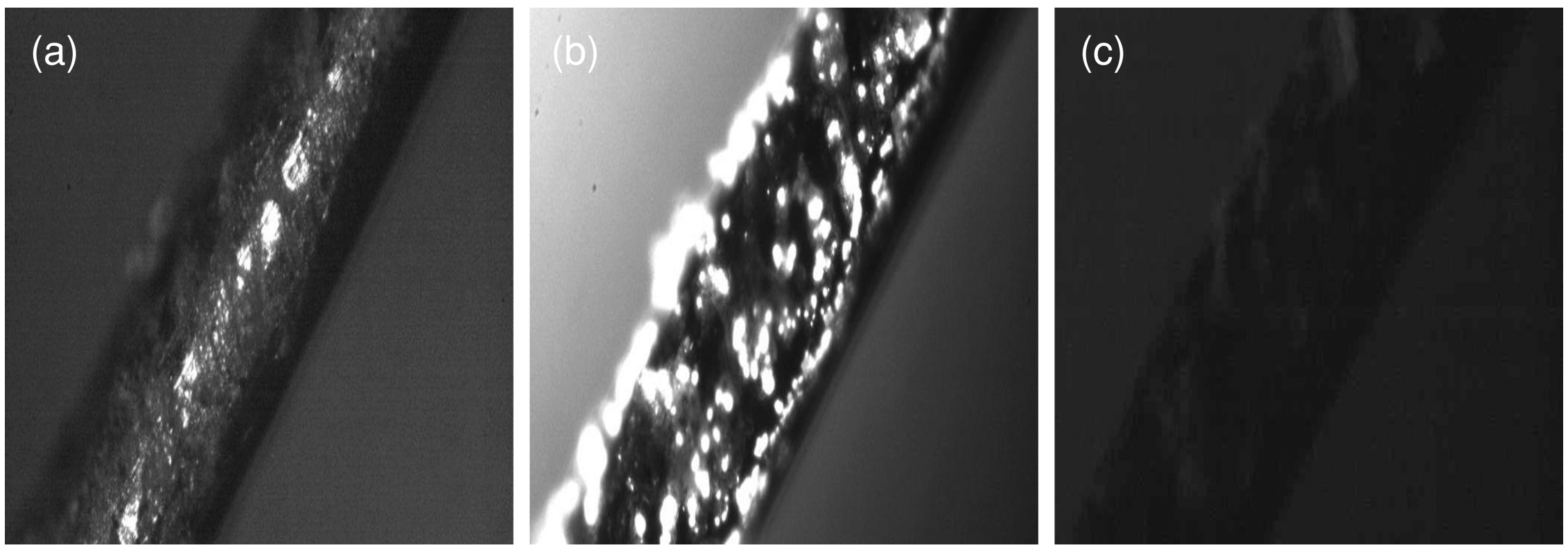

Figure S3: (a) White-light and (b) fluorescence image of a freshly dipped filament before the evaporation run. Fluorescence image of the same area on the filament after the evaporation run are shown in (c). The absence of fluorescence in (c) clearly shows that molecules have been successfully evaporated.

Table S1: Evaporation parameters. For the shown samples in the manuscript, different molecule evaporation parameters, including evaporation time, current through wire, vacuum level during evaporation, are displayed. The pre-cleaned wafers for samples 2 and 3 were first put in an acetone ultrasonic bath for 5 min, then rinsed with ethanol and isopropanol, and finally plasma-etched for 6 min, before transferring the sample.

Sample ID	Evaporation time	Wire current	Vacuum level	Transfer to a pre-cleaned Si-wafer
1	20 min	1 A	overpressure	no
2	1 min	0.9 A	$\approx$ 100 mbar	yes
3	1 min	1.07 A	$\approx$ 100 mbar	yes

1.2 Fabrication of hBN stacks

hBN flakes are first prepared on a clean silicon-wafer by dry-exfoliation [1] using an adhesive tape (Ultron Systems Inc., product number: 1007R-4.0), and then inspected under an optical

microscope. For the exfoliation, a small piece of a bulk hBN crystal (2D Semiconductors, product: BLK-hBN-LG) was transferred onto the adhesive tape and the remaining part was distributed over the whole adhesive area. Then, cut Si-wafer pieces (without pre-cleaning) were placed onto the tape and put onto a hotplate at 50 °C for 5 min. Afterwards, the tape is slowly removed over a period of a few minutes, while hBN flakes remain on the silicon chips. Then, potential candidates are chosen for the bottom and top flakes. The top flake is then picked up at a temperature of 130 °C with a polydimethylsiloxane (PDMS, product: SYLGARD 184 Silicone Elastomer Base + SYLGARD 184 Silicone Elastomer Curing Agent, 5:1 ratio) dome that is coated with a sacrificial polycarbonate (PC, HQ Graphene, product: 2D_CL_PC, 6% dissolved in chloroform) layer, which together form a "stamp". The PDMS domes are fabricated by putting droplets of the Base+Agent mixture onto microscope slides and placing them upside-down into an oven at 80 °C for 30 min. The domes then form under gravity. For PC, slides with thin PC layers are prepared by putting a droplet of the aforementioned PC solution onto them, after which another slide is placed on top, such that the liquid distributes over the whole slide area. Afterwards, the two slides are immediately separated from each other by quickly sliding the top slide away, resulting in a thin PC layer on the remaining slide. The subsequent stamp fabrication can be summarized as follows:

1. Pick a microscope slide with PDMS domes grown on them [Fig. **S4**(a)].

2. Take a piece of Kapton tape, cut a circular hole, and transfer PC from a microscope slide onto it, so that the hole is covered with PC [Fig. **S4**(b)].

3. Put the Kapton tape onto the PDMS dome, so that the circular hole containing PC aligns with the dome, resulting in a PDMS/PC stamp [Fig. **S4**(c)].

After fabricating the stamp, it is placed onto a hotplate and heated to 165 °C for 5 min. This relaxes the PC layer and results in a smoother surface, increasing the chances of a successful stacking. Then, the silicon chip that holds the bottom flake is put in the evaporation chamber. Perylene molecules are evaporated following the method described in section 1.1.

After evaporation, the silicon chip is mounted onto a copper stage that acts as a thermal

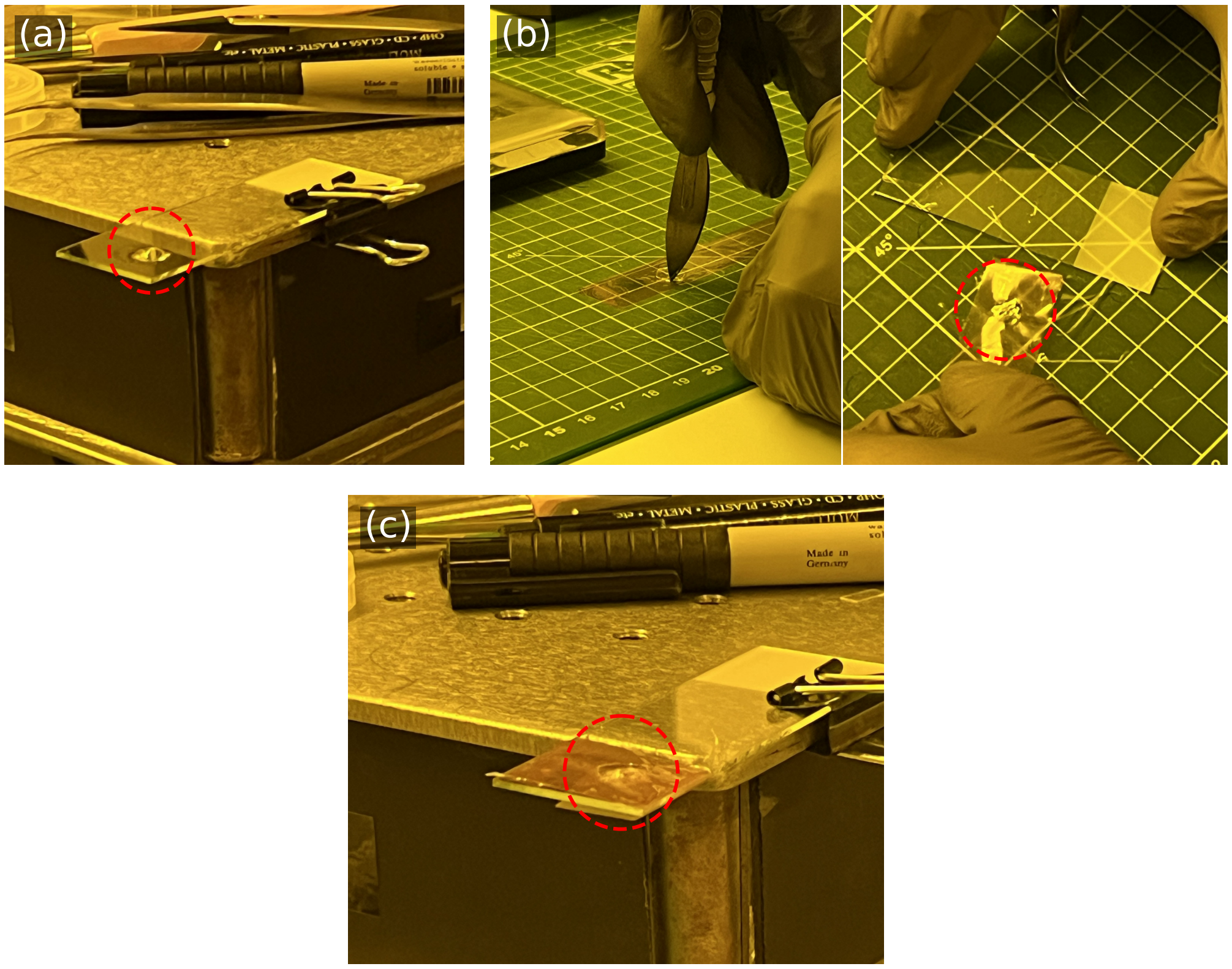

Figure S4: Stamp fabrication procedure. (a) PDMS dome grown on a microscope slide. (b) A circular hole is cut into a piece of Kapton tape with PC (which was spin coated onto another slide) transferred onto it afterwards. The hole is now covered with PC. (c) The Kapton tape is put onto the microscope slide, such that the PC-covered hole overlaps with the PDMS dome.

conductor. This stage is movable in the lateral direction with micrometer screws and its height can be adjusted using a step motor. The chip is fixed in the middle of the copper stage via a vacuum chuck. The slide carrying the stamp is then mounted on its own translational stage and is placed above the silicon chip. The copper stage is then heated to 130 °C before approaching the stamp. Right before the silicon chip and the stamp come into contact, the stage movement speed is set to the lowest possible speed of 0.14 μm/s, which is then kept during stacking. After the two hBN flakes are properly overlapped, a waiting time of approximately 10 min is taken to allow the PC layer to relax and thermalize properly with the silicon chip. From here, there are two possible paths of continuation:

1. **Direct delamination:** The stage is heated to 170 °C. Right before reaching this tem-

perature, the stage movement speed is set to 0.69 μm/s, and the stage is immediately driven out of contact. During the following delamination, a part of the PC layer detaches from the PDMS dome and remains on the silicon chip. The hBN stack and surrounding hBN flakes are now enveloped by a PC layer.

2. **Delamination on a clean chip after stack pickup:** The whole stack is first picked up and then transferred to a fresh silicon chip, which is cleaned in advance by putting it in an acetone ultrasonic bath for 5 min with subsequent rinsing with ethanol and isopropanol. After that, it is plasma-etched for 6 min. For the stack pickup, we keep a temperature of 130 °C. In this case, both the contact and the detachment speed of the stage are kept at 0.14 μm/s. The following transfer to the new silicon chip is done as described in point **1**.

In the general fabrication procedure, the PC layer is removed afterwards by immersing the chip in chloroform (Sigma Aldrich, product number: 32211-1L-M, purity $\geq$ 99.0%) for 30 min to 1 hour, followed by subsequent rinsing with acetone (VWR Chemicals, product number: 20066.321, purity $\geq$ 99.8%), ethanol (VWR Chemicals, product number: 20821.330, purity $\geq$ 99.8%) and isopropanol (VWR Chemicals, product number: 20880.320, purity $\geq$ 99.8%).

Figure **S5** shows optical microscope images of three samples used for measurements reported in the manuscript.

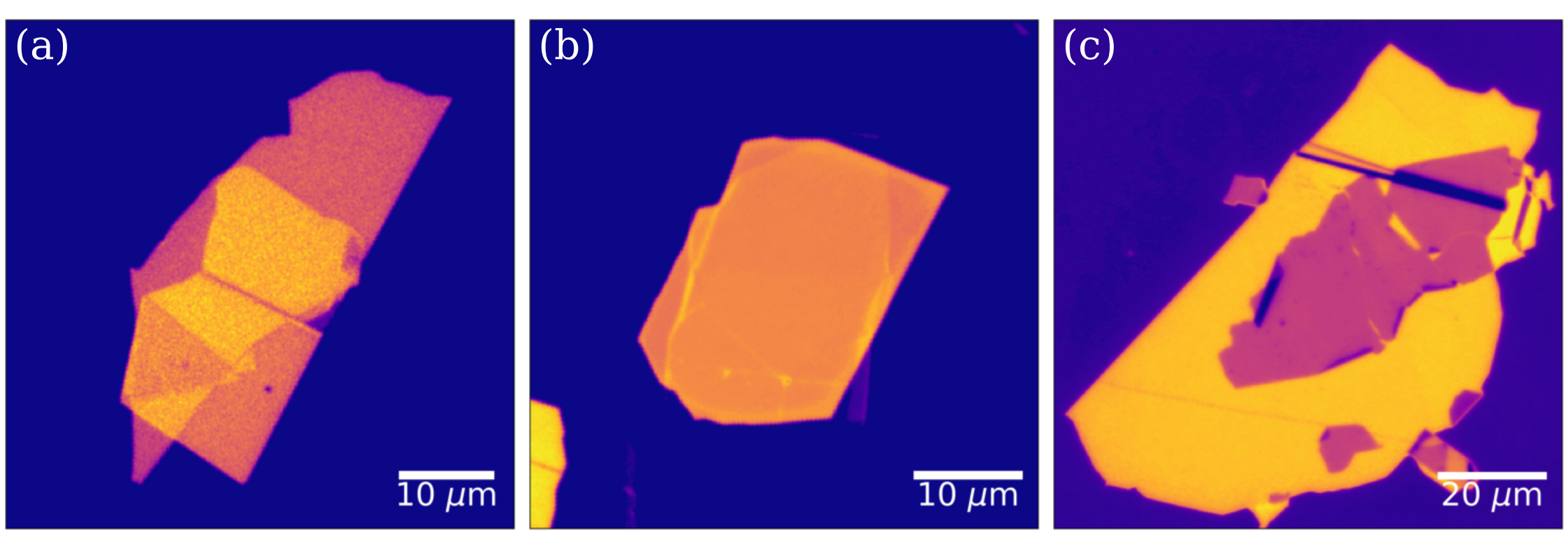

Figure S5: Overview of the three fabricated perylene/hBN samples investigated in this work. (a), (b), (c) show optical microscope images of Samples 1, 2, and 3, respectively.

1.3 Fluorescence images

Here, we relate the spectral data presented in the manuscript to the spatial locations of the corresponding emission sites on the respective samples. Fluorescence maps of the investigated samples are shown in Fig. **S6**. The overview images in Figs. **S6**(a),(d) correspond to Sample 1 and Sample 2, respectively. The selected regions indicate the sample areas from which the emission spectra, spectral diffusion traces, and resonance-excitation data shown in Figs. 2–4 of the manuscript were acquired. The sites of individual molecules are directly labeled in the fluorescence maps with red circles, and their correspondence to the data presented in the manuscript is summarized in Tab. **S2**.

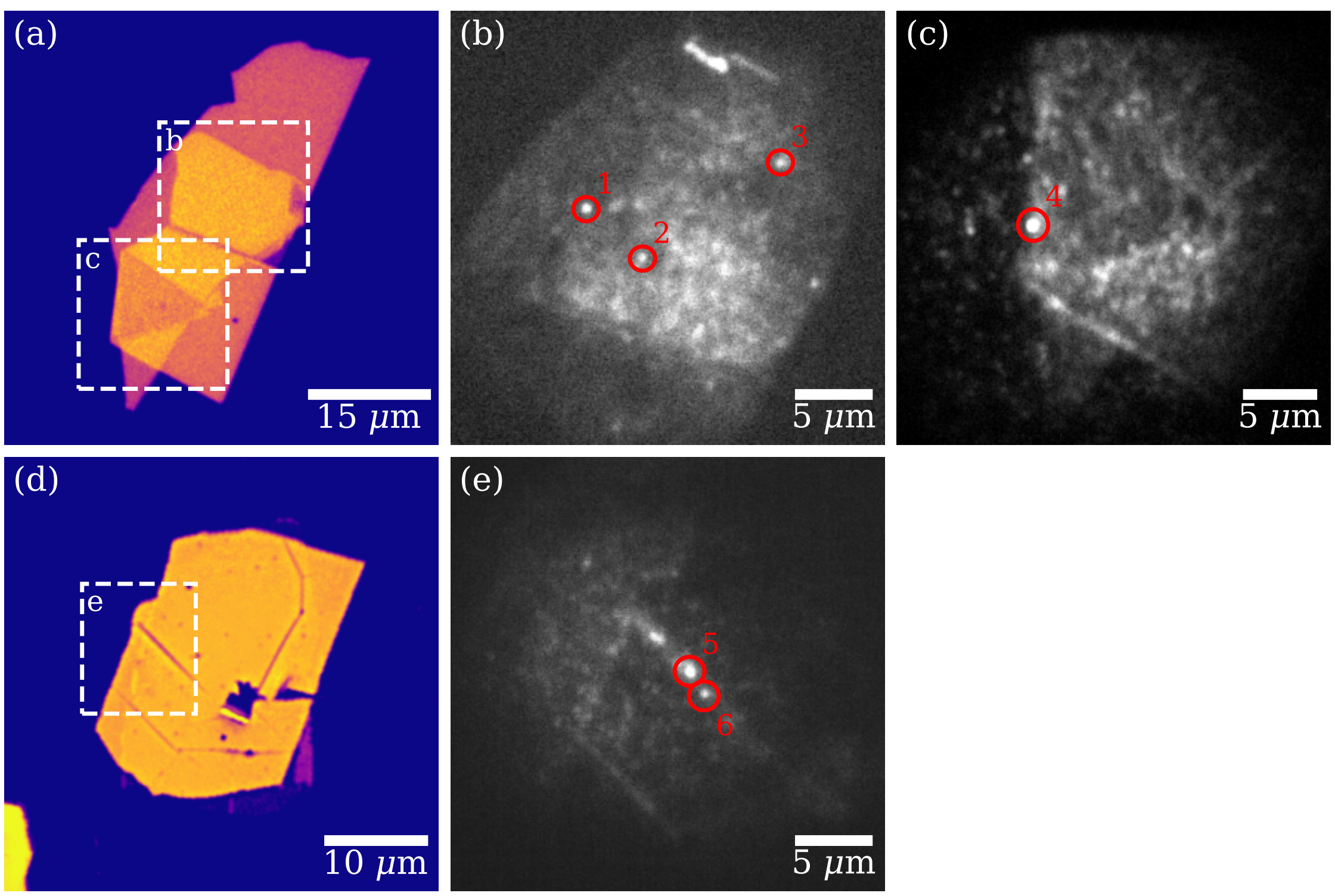

Figure S6: (a), (d) Overview microscope images of the investigated perylene/hBN samples. The white dashed boxes indicate the field of view shown in the corresponding enlarged fluorescence maps. (b), (c), (e) Fluorescence maps of the selected regions. The red circles indicate the individual molecular sites from which the spectral data were acquired.

Table S2: Correspondence between the spectral data shown in Figs. 2–4 of the manuscript and the measurement positions labeled in Fig. **S6**

Figure	Fluorescence Map
Fig. 2(a),(c)-i	(e)-5
Fig. 2(c)-ii	(b)-1
Fig. 2(c)-iii	(e)-6
Fig. 2(c)-iv	(b)-3
Fig. 3(a)	(b)-2
Fig. 3(c)	(e)-5
Fig. 4	(c)-4

1.4 Perylene in anthracene sample fabrication

The sample was prepared through sublimation of a mixture of perylene (Sigma Aldrich product number P11204) and anthracene (Sigma Aldrich, product number 10580, purity $>$ 99.0%). The anthracene material was additionally purified in a self-built zone refiner. The mixture had a mass ratio of perylene to anthracene of $1 : 2\,700\,000$.

A mass of 9.7 mg of mixture was put into a plasma-cleaned flask. The flask was evacuated and subsequently filled with around 1 atm of argon gas. A heat gun set to 400 °C was pointed underneath the flask, sublimating the mixture. Within three minutes crystals appeared in the flask. The flask was then emptied onto a petri dish from which a high-quality crystal was selected.

2 Experimental setup

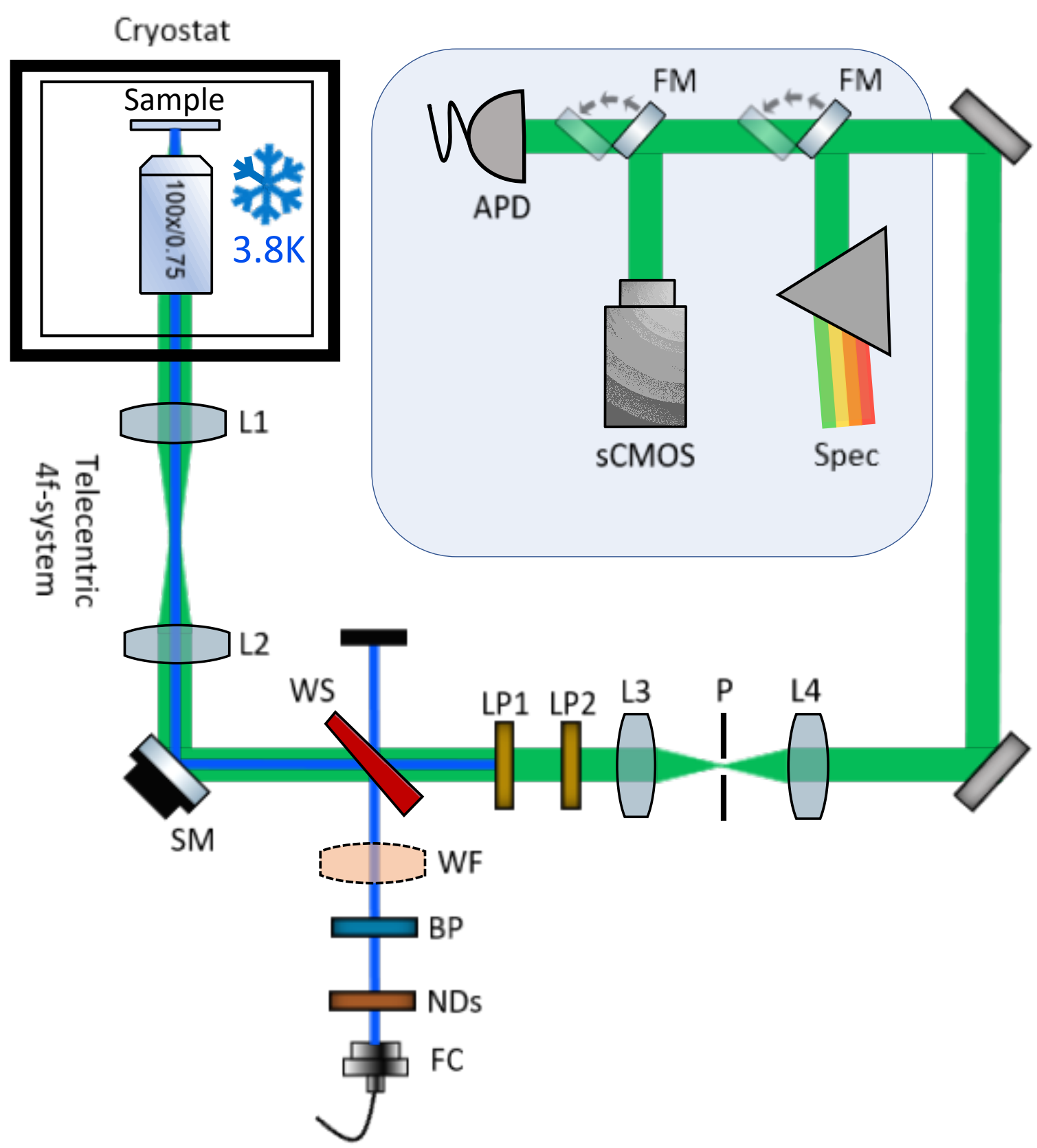

Figure S7: Sketch of the experimental setup. **L1–L4**: lenses. **BP** and **LP1–LP2**: bandpass and longpass filters. **SM**: steering mirror. **WF**: wide-field lens. **WS**: wedged sampler. **FC**: fiber collimator. **FM**: flip mirror. **sCMOS**: scientific CMOS camera. **Spec**: spectrometer. **APD**: avalanche photodiode. **P**: confocal pinhole. **NDs**: neutral-density filters.

The experimental setup sketched in Fig. **S7** allows us to perform single-molecule microscopy and spectroscopy at cryogenic temperatures below 4 K (Montana Instruments, CryoAdvance 100). For the illumination path, the excitation laser, which can be a diode laser at 454 nm (Toptica Photonics, DL pro HP 454), a diode laser at 423 nm (Omicron-Laser, LuxX 425–120), or the second-harmonic component of a Ti:Sapphire laser from 440 nm to 480 nm (Sirah Lasertechnik, Matisse CS–WaveTrain), is delivered through a single-mode fiber. The laser power can be attenuated in discrete steps using neutral-density filters. Afterwards, the laser passes through a bandpass filter to clean its spectral profile, and then it

encounters a wedged sampler, where 90% of the beam is transmitted and 10% is reflected. The reflected beam then propagates through a telecentric imaging system constructed by a 4f-lens system together with a steering mirror, enabling scan of the laser focus. After entering the cryostat, the laser is focused on the sample via an objective with a numerical aperture of 0.75 (Zeiss, LD EC Epiplan–Neofluar 100x/0.75 DIC M27). Furthermore, a wide-field lens can optionally be placed in front of the wedged sampler, allowing wide-field fluorescence imaging.

Fluorescence photons from the sample are collected by the same objective and guided backward through the common path before being directed to the wedged sampler, where 90% of photons are transmitted towards a set of longpass filters that filter out the excitation laser. The remaining fluorescence signal is focused through a confocal pinhole to reject out-of-focus fluorescence. Finally, signals are guided to various detection devices, including a low-noise sCMOS (Teledyne, Prime BSI), a spectrometer (Teledyne, SpectraPro HRS–300–S), and other detectors, depending on the measurement method.

3 Numerical calculations

The numerical study in this work involves three components. First, (time-dependent) density-functional theory (TDDFT) calculations are carried out using the ORCA software package.[2] The equilibrium ground-state geometry is obtained through structural relaxation employing the B3LYP exchange–correlation functional,[3,4] augmented by D4 dispersion corrections[5] and the def-TZVPD basis set. Starting from these optimized geometries, we compute excited-state properties using Casida's linear-response time-dependent DFT formalism,[6] including the Tamm–Dancoff approximation. The (TD)DFT calculations provide access to the relaxed molecular structure in the S_0 and S_1 manifolds that are required to estimate the Huang-Rhys factors.

The second step addresses the phononic structure of the extended host. Our simulations address configurations in which perylene is incorporated either between extended AA'-stacked hBN slabs that deform in response to the molecule, or at extended AA'-stacked defective structures, such as edges and steps. Density-functional theory becomes costly for such systems that involve thousands of atoms. Instead, we model molecular motion using the ASE software package[7] in combination with the foundation machine-learning interatomic potential MACE-MP (medium size)[8] and explicitly account for dispersive interactions.

Thirdly, we utilize MACE-MP in combination with structure relaxation to gain a microscopic understanding of the possible binding-sites of perylene. All cell parameters and nuclear positions are relaxed for 50 000 steps using the FIRE optimization algorithm.

3.1 Visualization of insertion geometries

In this section, we plot the insertion geometries of all numerical calculations presented in Tab. 2 of the manuscript. The calculations were performed for a single perylene molecule embedded in various hBN structures. For each structure, the formation energy is calculated as $E_f = E_{\mathrm{Mol+hBN}} - (E_{\mathrm{Mol}} + E_{\mathrm{hBN}})$, where $E_{\mathrm{Mol+hBN}}$ is the total energy of the combined

molecule-hBN system, and E_{Mol} and E_{hBN} are the energies evaluated with MACE-MP of separately relaxed molecule and hBN structure, respectively. The formation energy therefore describes how stable the formation of a bound perylene-hBN system is. The corresponding structures E1 - E16 in Tab. 2 of the manuscript are shown in the Figs. **S8** - **S12**. For views from the top, several layers have been omitted for visualization purposes.

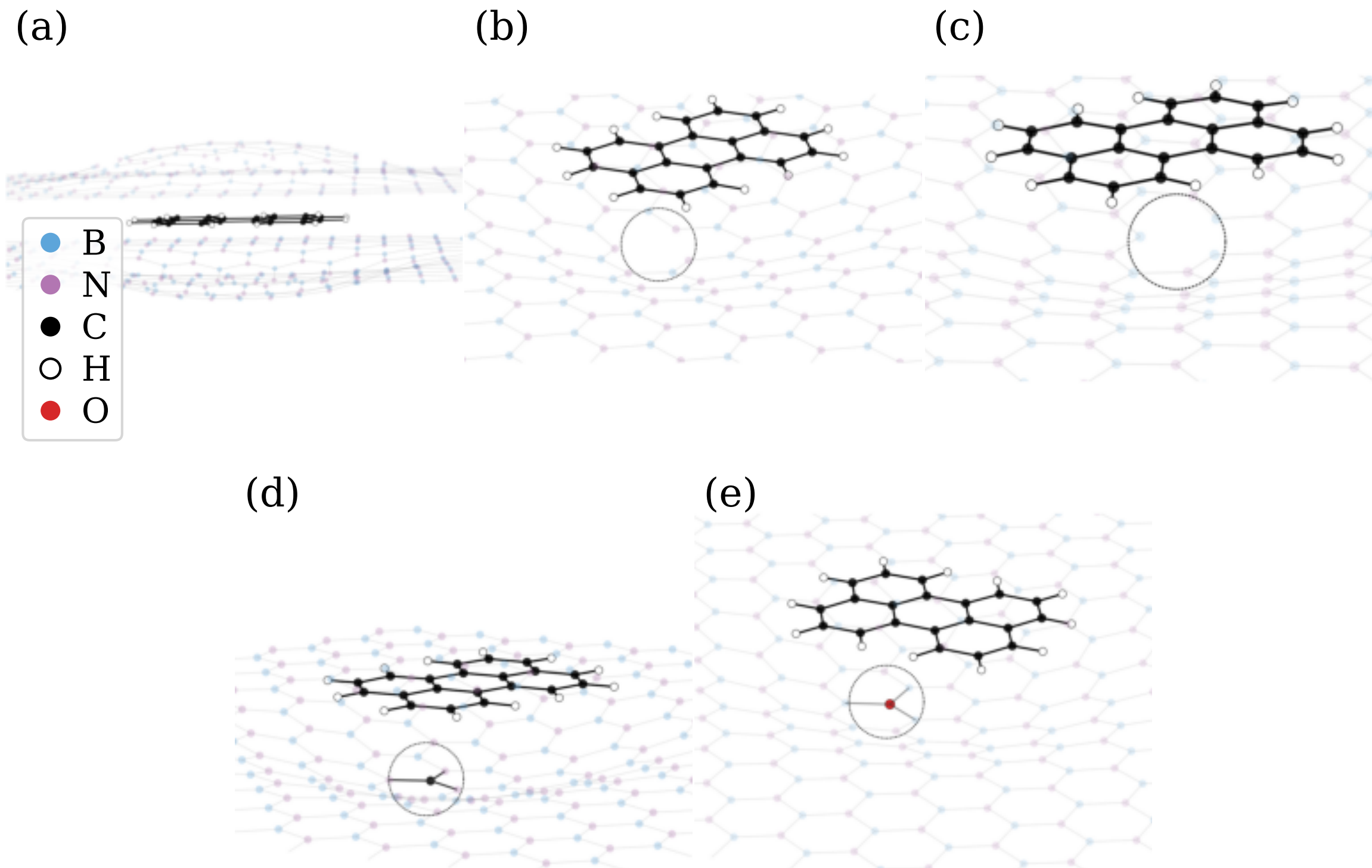

Figure S8: Perylene in an hBN bilayer stack (E1–E5). (a) The side view for perylene in the pristine structure. This side view is identical for the other structures in this figure as well. (b-e) Close-ups into the inside of the stack, with different visible substitutional point defects (inside black circles with dashed lines), where (b) and (c) show B- and N-vacancies, respectively. For these images, the top layer has been omitted for visualization purposes.

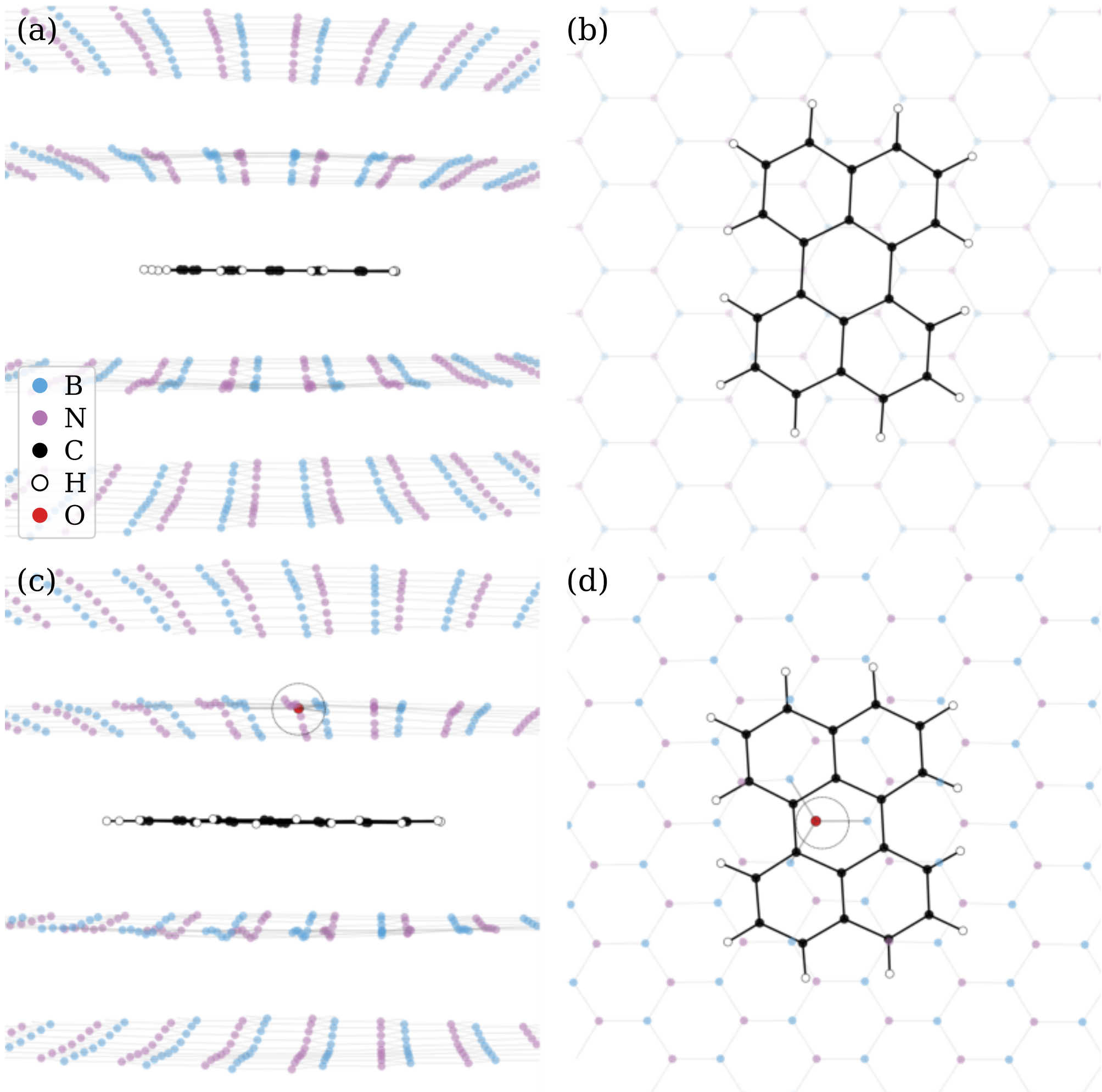

Figure S9: Perylene in an hBN multilayer stack (E6–E7). (a), (b) Perylene side and top views in the pristine structure . (c), (d) Perylene side and bottom views in an hBN structure containing an oxygen point defect. For the top views in (b) and (c), most top and bottom layers have been omitted for visualization purposes.

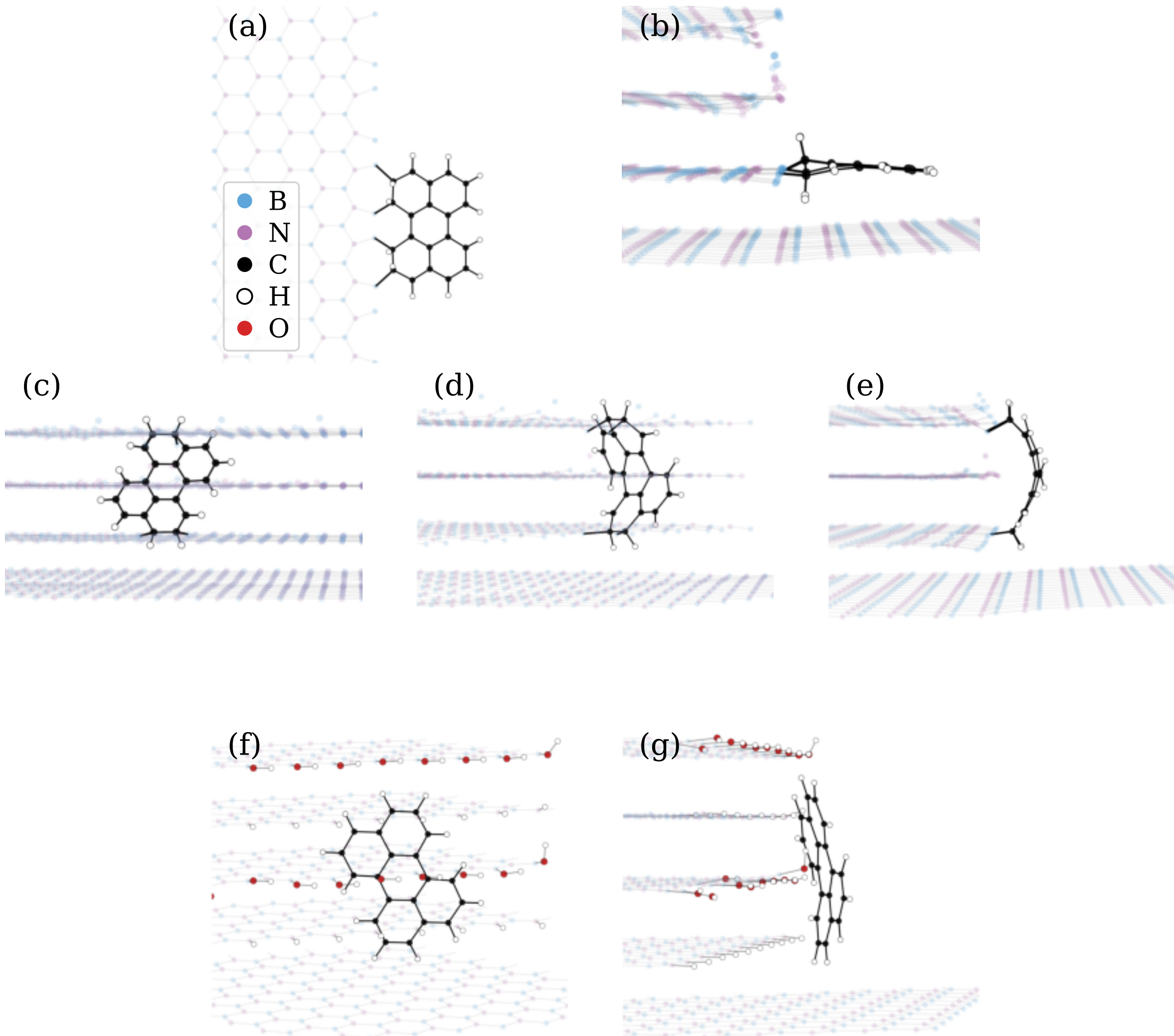

Figure S10: Perylene in an hBN multi-step edge structure (E8–E10). (a), (b) Perylene (“lying”) top and side views in the pristine structure. One can see B and N atoms forming covalent interlayer bonds in some parts of the edge. (c-e) Perylene (“standing”) front and side views in the pristine structure. (f), (g) Perylene (“standing”) front and side views in an OH/H-terminated structure. For the top view in (a), most layers have been omitted for visualization purposes. Structures E8 and E9 contain covalent bonds between the molecules' C atoms and B atoms.

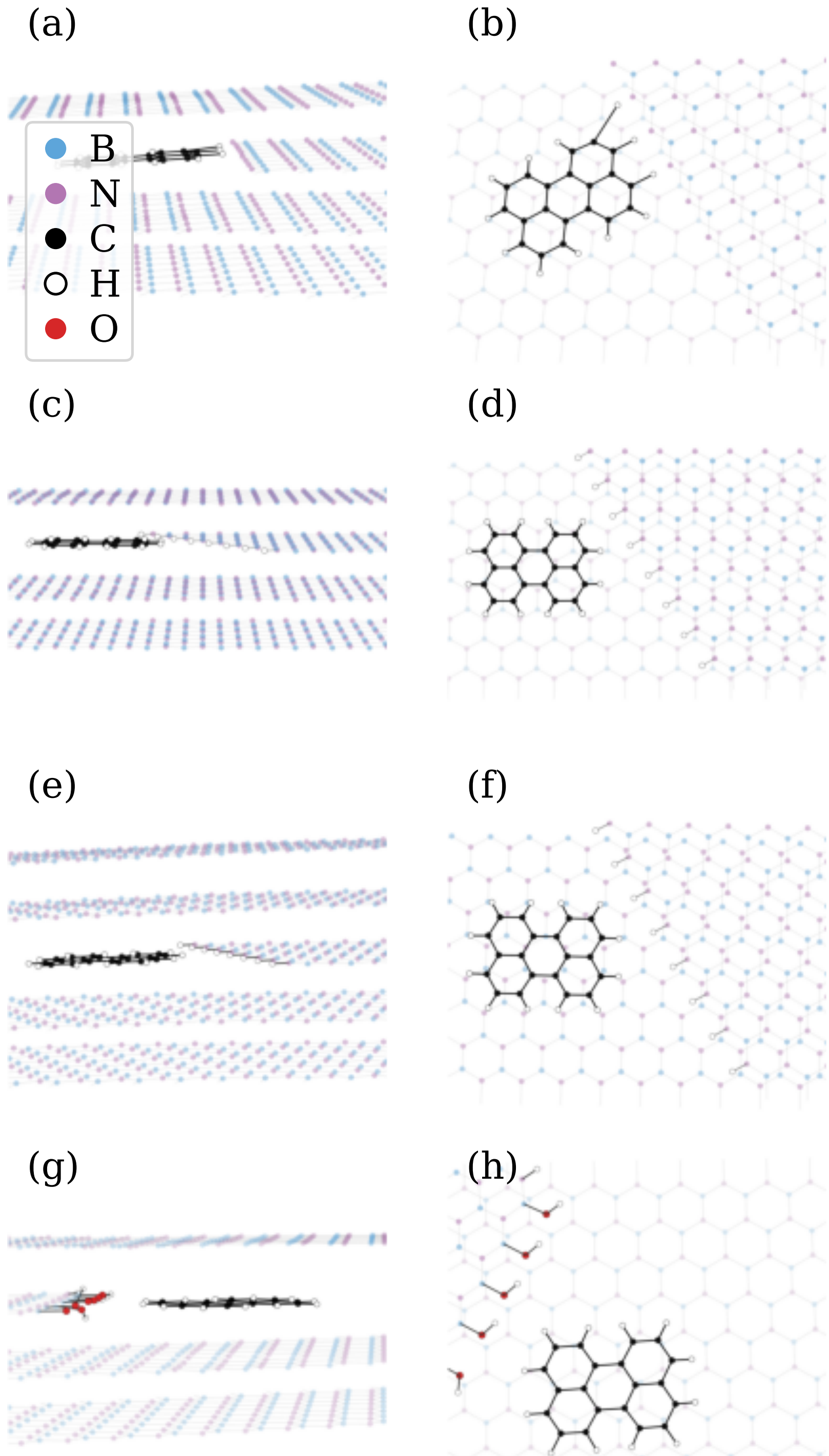

Figure S11: Perylene in an hBN embedded step edge structure (E11–E14). (a), (b) Perylene side and top views in the pristine structure. (c), (d) Perylene side and top views in a H-terminated structure. (e), (f) Perylene side and top views in a larger H-terminated structure, containing an additional hBN layer. (g), (h) Perylene side and top views in an OH-terminated structure. For the top views, most layers have been omitted for visualization purposes.

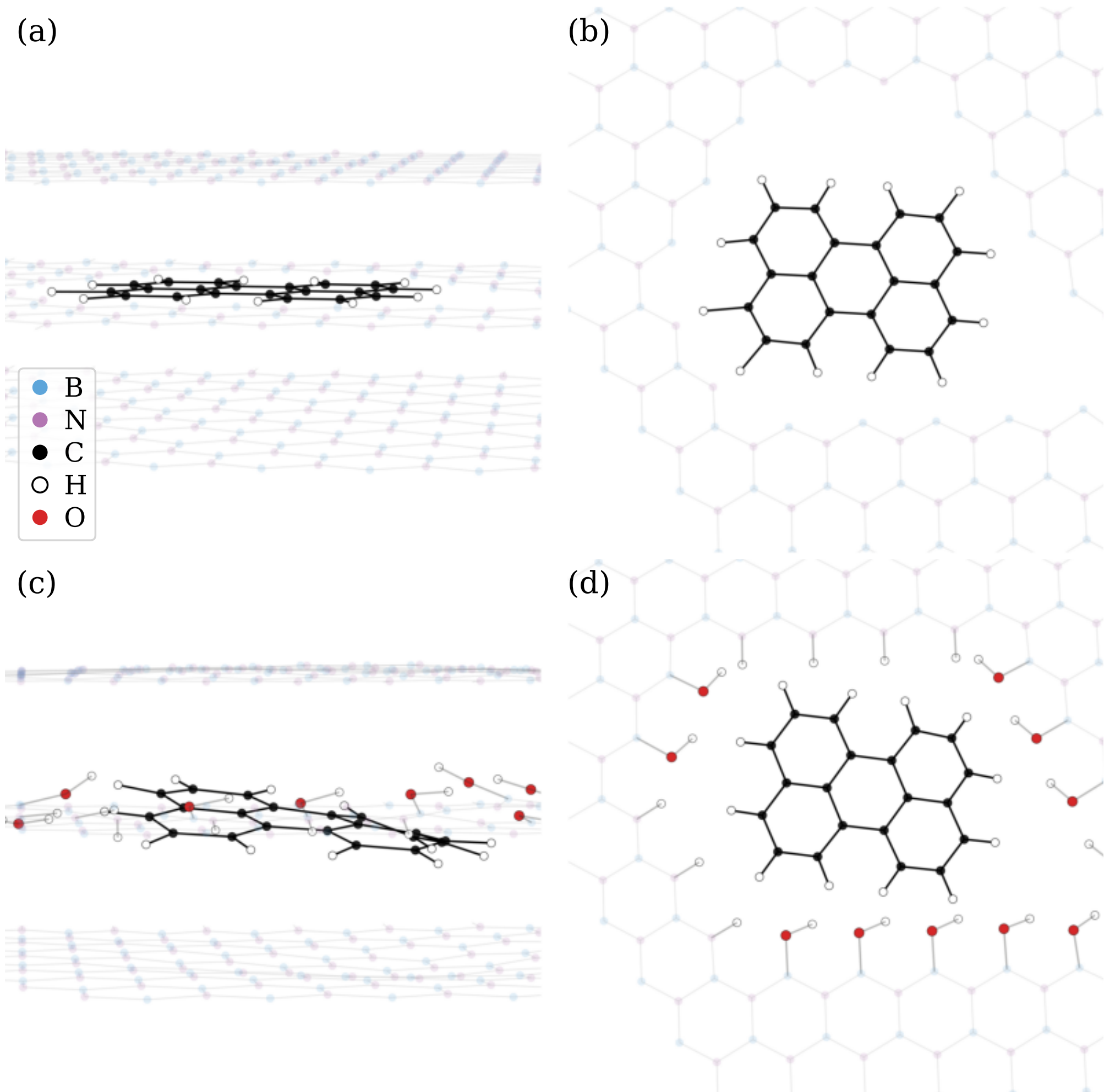

Figure S12: Perylene in an hBN extended vacancy structure (E15–E16). (a), (b) Perylene side and top views in an unterminated vacancy structure. (c), (d) Perylene side and top views in a H- and OH-terminated vacancy structure. For the top views, most layers have been omitted for visualization purposes.

3.2 Diffusion barrier of perylene in an hBN stack

Fig. **S13** displays the relative enthalpy of the perylene/hBN system when the molecule is displaced by up to one hBN lattice in the stack. The geometry of the molecule is held fixed while the hBN stack is allowed to relax after each step. It can be seen that a plateau is reached after a displacement of around 1.8 Å, which acts as an upper bound in the energy needed to displace the perylene molecule from one lattice site to the next. Since this energy is comparable to the thermal energy at room temperature (25.7 meV at 300 K), the associated Boltzmann factor is therefore $\exp(-54.6\,\mathrm{meV}/25.7\,\mathrm{meV}) \approx 0.12$, which indicates that thermally activated hopping between neighbouring hBN lattice sites is plausible at room temperature. The hopping yield would further increase during the stacking and delamination process, when the stack is heated up to around 170 °C, and the resulting Boltzmann factor is $\exp(-54.6\,\mathrm{meV}/38.2\,\mathrm{meV}) \approx 0.24$. In addition, the upper stack is approached onto the lower flake at an inclined angle. This geometrical factor should also assist perylene molecules to be progressively expelled from the pristine stack.

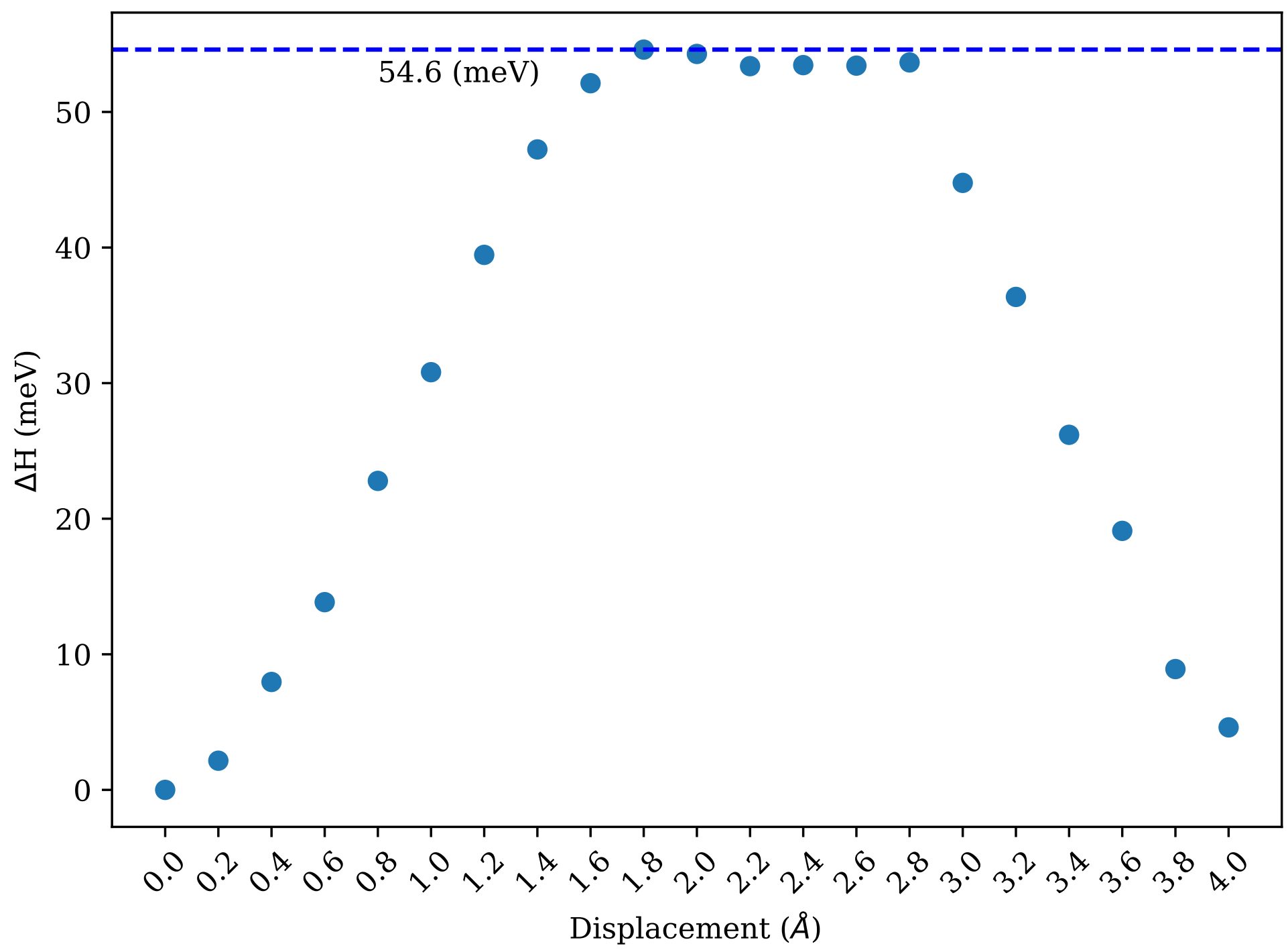

Figure S13: Relative enthalpy of perylene in an hBN stack versus the displacement of perylene from one lattice site to another. It can be seen that the diffusion barrier is around 54.6 meV. After reaching the next lattice site, the relative enthalpy decreases again, until it reaches the next minimum.

4 Emission spectra

In this section, we present the Franck-Condon simulation algorithm performed for perylene in hBN, together with the phonon-broadening model used to account for the coupling to lattice vibrations. The resulting vibronic spectra are then compared with the experimentally measured emission spectra of perylene in hBN, as shown in Fig. 2(c) of the manuscript.

4.1 Calculation of Franck-Condon factors

Starting from relaxed ground- and excited-state geometries, vibronic spectra were calculated within the displaced harmonic oscillator approximation. The electronic ground and excited potential energy surfaces are assumed to be harmonic and to share identical normal modes.

Normal modes were expressed in mass-weighted Cartesian coordinates. The displacement between the ground- and excited-state equilibrium geometries of the molecule, $\mathbf{\Delta R}$, is projected onto each mass-weighted normal mode $\mathbf{e}_{ij}$ (where i is an index describing the number of atoms with their 3 degrees of freedom and j describes the number of all modes) to obtain the normal-coordinate displacements

$$\Delta Q_j = \sum_i \sqrt{m_i}\, \mathbf{e}_{ij} \cdot \mathbf{\Delta R}_i. \tag{1}$$

Here, m_i denotes the atomic masses, and the index i in this case runs over all perylene atoms (and their respective 3 degrees of freedom, so 96 in total). For our simulations, the resulting ΔQ_j is now a vector with length equal to the total number of modes (coming from all atoms in the hBN-structures + 32 perylene atoms, each with their 3 degrees of freedom). From this, we calculate the Huang-Rhys factor for each mode j.[9]

$$S_j = \frac{\omega_j}{2\hbar}(\Delta Q_j)^2, \tag{2}$$

with $\omega_j = 2\pi c\, \nu_j$, where ν_j is the vibrational wavenumber.

Within the harmonic approximation, the Franck-Condon (FC) overlap between the vibrational ground state and the v_m-th excited state of mode m follows a Poisson distribution,[10]

$$P_m(v_m) = e^{-S_m}\, \frac{S_m^{v_m}}{v_m!}. \tag{3}$$

In order to decrease the calculation time, a threshold for the FC-overlaps is set, in order to filter out modes that do not have any significant contribution. With the resulting "active modes", the rest of the simulation was performed.

Multimode vibronic transitions were constructed by enumerating all combinations of vibrational quantum numbers $\{v_m\}$ up to a maximum total vibrational excitation $v_{\max}$ (with

$v_{\max} \geq \sum_m v_m$). Each transition contributes with a wavenumber shift

$$\Delta\nu = \sum_m v_m \nu_m \tag{4}$$

with a corresponding Franck-Condon weight

$$W(\{v_m\}) = \prod_m P_m(v_m). \tag{5}$$

This procedure creates a discrete spectrum, as seen in Fig. **S14**(a). For this specific calculation, only 6 active modes contribute to the spectrum. The occupation quantum numbers for each peak numbers are shown on the top axis of the figure.

4.2 Phonon broadening model

After calculating the Franck-Condon weights for the respective transitions, a broadening model was used to model phonon broadening of each Franck-Condon transitions. Simulation peaks that were very close to each other (distance of up to 5 cm^{-1}), were binned together, so that many energetically close peaks are replaced by one stronger peak.

The model assumes that every peak can be split into a narrow zero-phonon contribution (linewidth limited by the spectrometer resolution) and a broader phonon-sideband contribution due to Debye-Waller coupling, inspired by Grushka[11] :

$$S(\nu) = \sum_k w_k \left[\alpha_{\mathrm{DW}}\, G(\nu; \nu_k, \sigma_k) + (1 - \alpha_{\mathrm{DW}})\, \mathrm{EMG}(\nu; \nu_k, \sigma_k, \tau_{\mathrm{ph}})\right], \tag{6}$$

where G is a unit-area Gaussian and EMG is a unit-area exponentially modified Gaussian (Gaussian convolved with a one-sided exponential tail with decay length $\tau_{\mathrm{ph}} = 70\,\mathrm{cm}^{-1}$). w_k are the weights of the corresponding Franck-Condon bars and α_{DW} is the mean Debye-Waller factor obtained from the measured spectra using $\alpha_{\mathrm{DW}} = I_{\mathrm{ZPL}}/(I_{\mathrm{ZPL}} + I_{\mathrm{Phonon}})$. Here, we determine a mean value of $\alpha_{\mathrm{DW}} = 0.49 \pm 0.01$. For σph, a global ZPL linewidth of

9.5 cm^{-1} is used for every peak, which, for our emission spectra, is limited by the resolution of the spectrometer. This global linewidth is obtained by fitting a Gaussian to the 00-ZPL.

In Fig. **S14**(b), a broadened spectrum can be seen. Here, the discrete spectrum in Fig. **S14**(a) is used for the broadening procedure. The red dashed line shows the point after which the whole broadened spectrum has been scaled up by a factor for visualization.

4.3 Temperature-dependent emission spectra

Fig. **S15** shows the emission spectra of three perylene sites at different temperatures. As the temperature increases from 4 K, phonon coupling becomes more pronounced, and the initially narrow ZPL and vibronic peaks gradually broaden and lose contrast. Starting around 55 K, the ZPLs are overwhelmed by their respective phonon sidebands. The peak between 440 and 443 nm maintains a constant profile, regardless of measurement position on the sample. The amount of red shift from the excitation wavelength 423.3 nm, which is 900 to 1000 cm^{-1}, matches the second-order Raman scattering from silicon.[12] The first-order Raman scattering expected at 520 cm^{-1} is blocked by the longpass filter.

Several experimental factors should be considered when interpreting this series of measurements. On the one hand, when the temperature is increased, not only does the linewidth of perylene molecules broaden, but the transition peaks of other organic residues also broaden. Thus, they are more likely to be excited to fluoresce at higher temperatures, affecting the distinguishability of the target molecules and limiting further measurements. On the other hand, photobleaching occurs more readily at higher temperatures, especially when the temperature approaches 77 K. In addition, the temperature changes cause spatial drift of the sample, as the sample is mounted on a nano-positioner. Consequently, the lateral position and focal length of the objective need to be readjusted after each temperature change, introducing variations in the illumination and photon collection conditions.

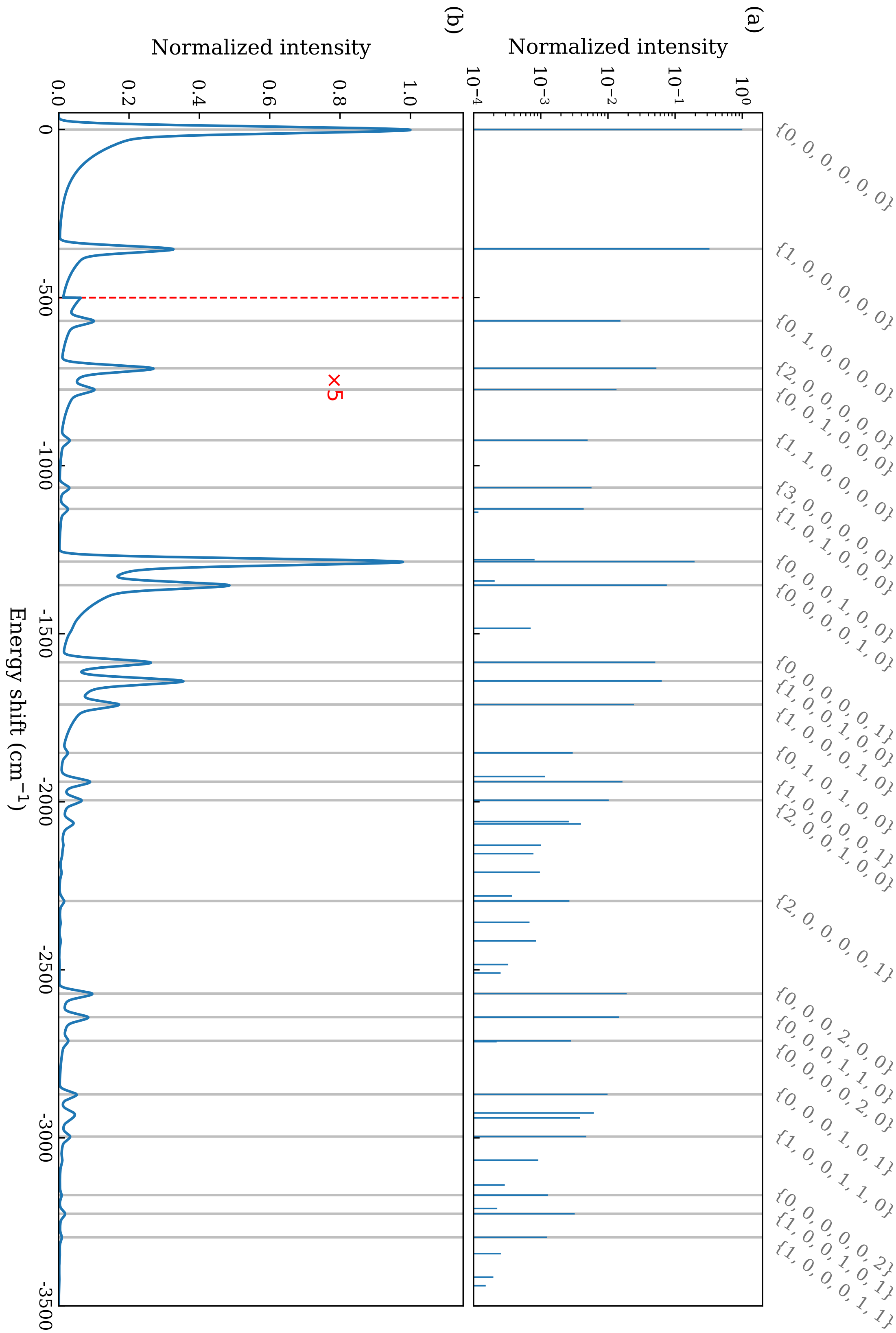

Figure S14: (a) Franck-Condon simulated spectrum. (b) Phonon-broadening model applied to the spectrum in (a).

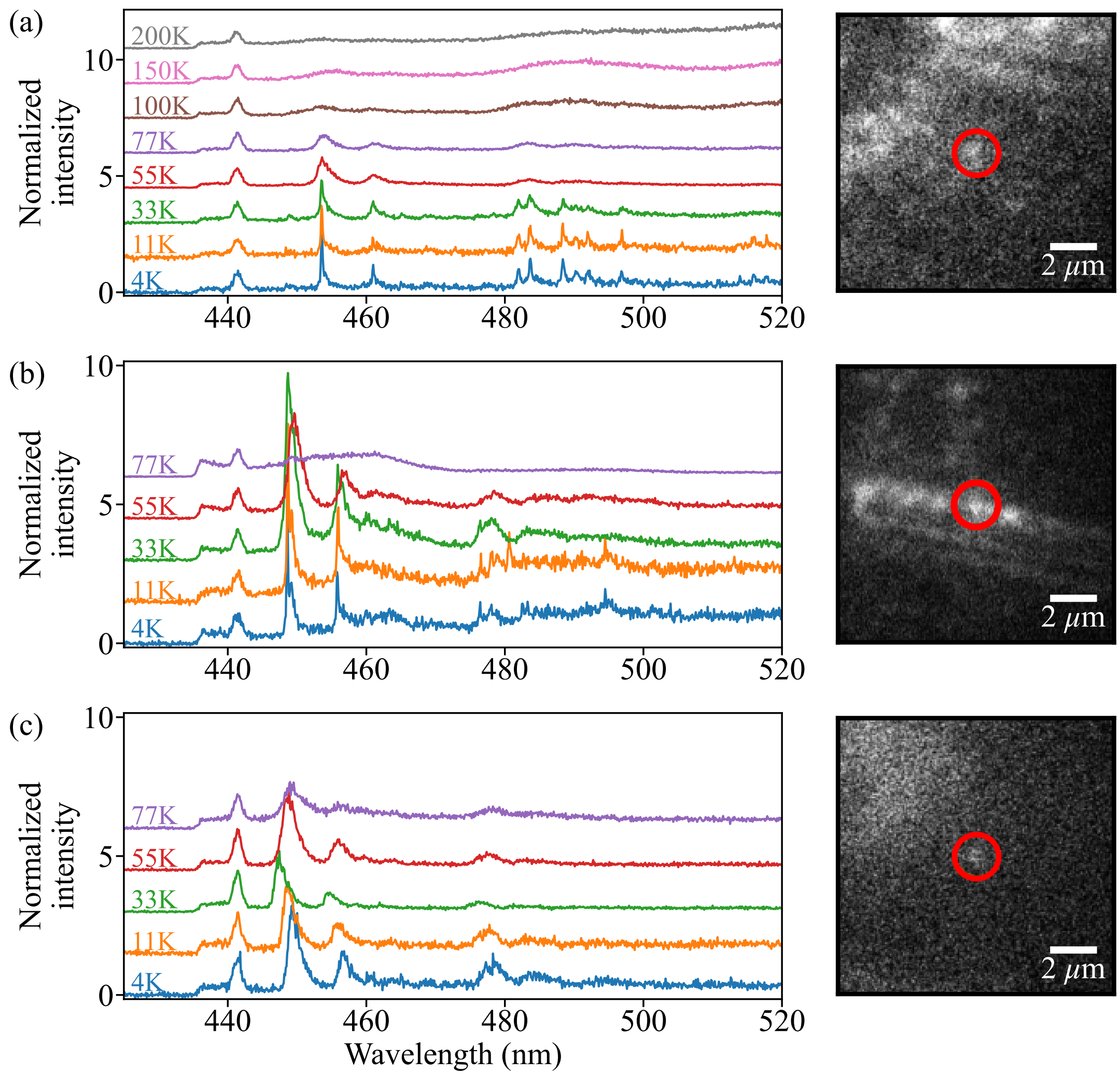

Figure S15: Temperature-dependent fluorescence spectra. (a)–(c) Spectra of individual perylene emission sites in the hBN stack recorded at various temperatures, showing thermal broadening of the spectral features. The right panels show the corresponding fluorescence images, with the measured emitters marked by red circles. All spectra are vertically offset for clarity.

5 Spectral diffusion data analysis

In this section, we provide more detailed analysis and discussion of the spectra presented in Fig. 3(a) of the manuscript. The complete spectral trace measured on a diffraction-limited spot over a time window of 400 s is plotted in Fig. **S16**. Almost all resolvable peaks can be assigned to individual molecules by comparing with the vibronic fingerprint of perylene. We

identify in total six distinct molecular emission traces, denoted as Molecule 1 - Molecule 6, and corresponding digits in Fig. **S16**(b). Limited by the sensitivity of the spectrometer, only the ZPL and up to two vibronic peaks could be reliably identified for Molecule 1 - Molecule 4. In contrast, Molecule 5 and Molecule 6 exhibit a more complete vibronic fingerprint.

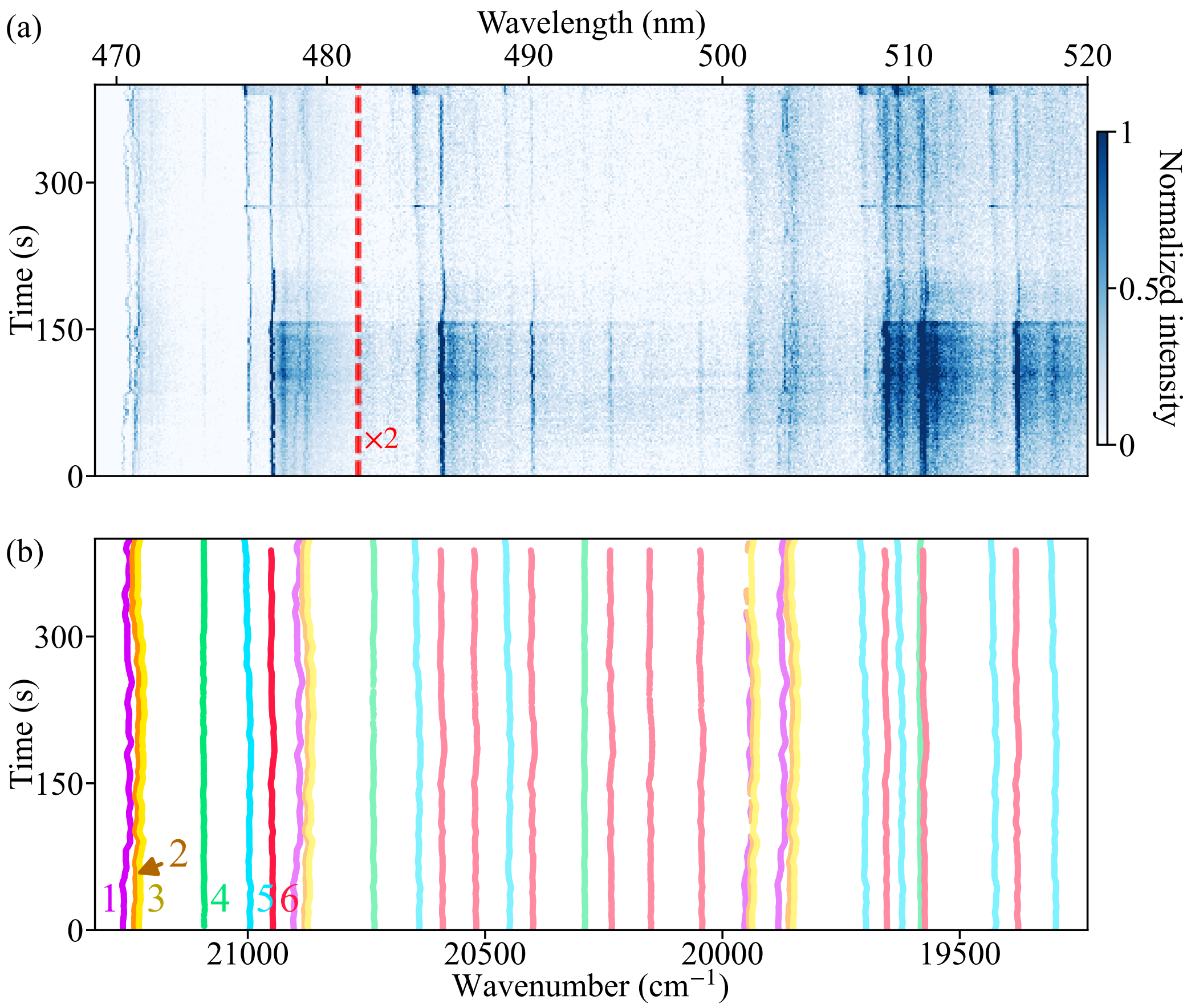

Figure S16: Spectral evolution of six molecules within one diffraction-limited spot. (a) Time-resolved emission spectra recorded over 400 s. (b) Extracted spectral diffusion traces of the identifiable ZPL and vibronic peaks of six molecules. Peaks belonging to the same molecule are labeled by the same color. The bold lines denote the ZPL transitions, and the narrow lines are the vibronic transitions.

The traces of all identifiable ZPL and vibronic peaks are presented in Fig. **S16**(b). For each of the molecules, the ZPL and its vibronic peaks follow the same spectral diffusion trajectory, indicating that the full vibronic fingerprint shifts synchronously. The synchronized drift further reinforces the assignment of these peaks to the same single molecule and sug-

gests that the dominant perturbation primarily shifts the entire electronic transition, while leaving the internal energy spacing of the associated vibronic states unchanged.

To gain further insights, we extract three time-dependent observables for each molecule: the spectral diffusion trajectory $\Delta\nu(t)$, the ZPL peak intensity $I_{\mathrm{ZPL}}(t)$, and a relative ZPL weight $w_{\mathrm{ZPL}}(t)$ defined with respect to a fixed set of vibronic peaks defined as $w_{\mathrm{ZPL}}(t) = A_{\mathrm{ZPL}}/(A_{\mathrm{ZPL}} + A_{6\mathrm{fv}})$. Here, A_{ZPL} is the integral over a 10 cm^{-1} width window centered on the ZPL position, and $A_{6\mathrm{fv}}$ is the sum of the integrated spectral window based on the six fundamental vibrational modes plotted in Fig. 2(d) of the main manuscript. The resulting time traces for the three observables are presented in Fig. **S17**.

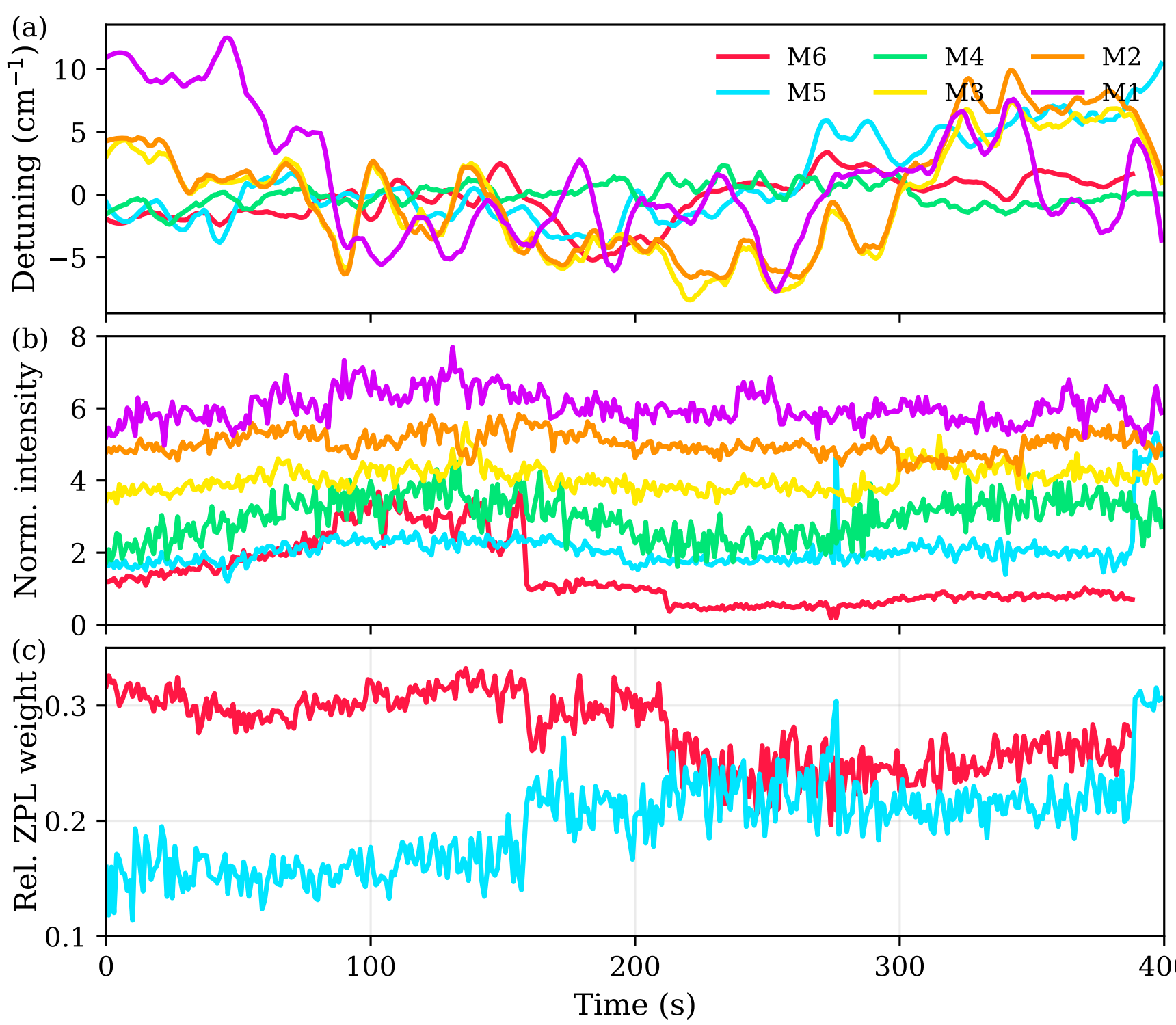

Figure S17: Extracted three time-dependent observables for the six molecules. (a) Spectral diffusion trajectories of the six molecules. (b) Median-normalized ZPL peak heights of molecules, vertically offset by constant amounts for clarity. (c) Defined relative ZPL weight for Molecule 5 and Molecule 6.

Fig. **S17**(a) shows the diffusion temporal trajectories. Among the six molecules, only Molecule 2 and Molecule 3 follow the same spectral diffusion pattern. The remaining molecules do not show obvious mutual correlation in their frequency trajectories. In Fig. **S17**(b),

the relative variations of ZPL emission intensity $I_{\mathrm{ZPL}}(t)$ are presented after normalization to their median value, and applying a constant vertical offset for better visualization. The ZPL intensity remains relatively stable for Molecule 1-Molecule 5 around their respective baselines. By contrast, Molecule 6 alone shows pronounced intensity variation, indicating a molecule-specific process rather than a global effect across the diffraction-limited spot. A reasonable interpretation is that Molecule 6 undergoes a local environment change, possibly local charge-state fluctuations,[13] that modifies its emission efficiency. Furthermore, in the later stage, the intensity of Molecule 6 completely vanishes, while Molecule 5 shows a strong increase at the same time. This anti-correlation appears to be specific to the Molecule 5-Molecule 6 pair and may indicate some degree of mutual coupling. However, whether it reflects an energy-transfer-like process cannot be determined confidently by the present data.

As shown in Fig. **S17**(c), the relative ZPL weight $w_{\mathrm{ZPL}}(t)$ of Molecule 5 and Molecule 6 exhibits stage-like behavior. Molecule 5 shows a three-step evolution from a lower-w_{ZPL} to a higher-w_{ZPL} regime. Molecule 6, in contrast, starts from a relatively high w_{ZPL} level, undergoing a clear but transient reduction at intermediate times, and ends at a further lower w_{ZPL} stage. Such behavior suggests that these two molecules may possess a small number of metastable local configurations associated with changes in the ZPL-vibronic branching ratio.[14,15] It should be noted that $w_{\mathrm{ZPL}}(t)$ is not fully independent of the peak height $I_{\mathrm{ZPL}}(t)$, because $I_{\mathrm{ZPL}}(t)$ significantly contributes to A_{ZPL}. Accordingly, an increase in $I_{\mathrm{ZPL}}(t)$ can raise $w_{\mathrm{ZPL}}(t)$. However, this mathematical effect does not fully account for the behavior observed in Molecules 5 and 6. For Molecule 5, the increase in $w_{\mathrm{ZPL}}(t)$ occurs while $I_{\mathrm{ZPL}}(t)$ remains relatively stable or even slightly decreases over the same period. This implies that the rise of $w_{\mathrm{ZPL}}(t)$ in Molecule 5 points to a suppression of the vibronic peak contribution. For Molecule 6, the variations in $I_{\mathrm{ZPL}}(t)$, including both its gradual increase and two rapid drops, do not lead to a synchronous change in $w_{\mathrm{ZPL}}(t)$. This suggests that the large intensity modulation in Molecule 6 is not directly tied to a long-term redistribution of the relative ZPL spectral weight.

Overall, although the present data do not uniquely identify the microscopic origin of the spectral evolution, they support a picture in which the observed emission dynamics arise from both continuous molecule-specific local perturbations and occasional switching between a small number of metastable local configurations.

6 Sample Annealing

In this section, we discuss the influence of high-temperature baking on the spectral stability. Here, we used Sample 2 in which perylene molecules had already been encapsulated in the hBN stack and baked it in air at 750 °C for 4 h. We expected this process to remove small trapped molecules, such as oxygen and water, from the stack even after encapsulation, and thereby potentially improve the spectral stability of perylene.

The optical microscope and AFM images after annealing are shown in Figs. **S18**(a),(b). Compared with the original images before annealing shown in Fig. **S5**, the two hBN layers can still be resolved, indicating that the overall flake geometry is preserved after annealing. However, additional cracks and dot-like features appear within the hBN layers. These structural changes may originate from thermally induced stress, possibly related to the mismatch in thermal expansion coefficients between hBN and the silicon substrate. In addition, a region with strong optical contrast appears within the hBN flake. In the corresponding AFM image, this area shows a pronounced topographic contrast, indicating local damage or deformation of the hBN layer.

Optical measurements suggest that annealing improves the spectral stability of perylene. As shown in Fig. 3(c) of the manuscript and Fig. **S18**(d), on the annealed sample, the emission traces of perylene molecules are much more stable, with only occasional drifts in frequency and intensity over several minutes. This is a proof that removing small molecules from the stack significantly removes the noise seen by perylene molecules, and these noise sources could be due to bond-flips or change of charge states in these unwanted molecules.

Under efficient resonant excitation, stable emission remains for up to around 20 minutes. At later times, they may exhibit photobleaching.

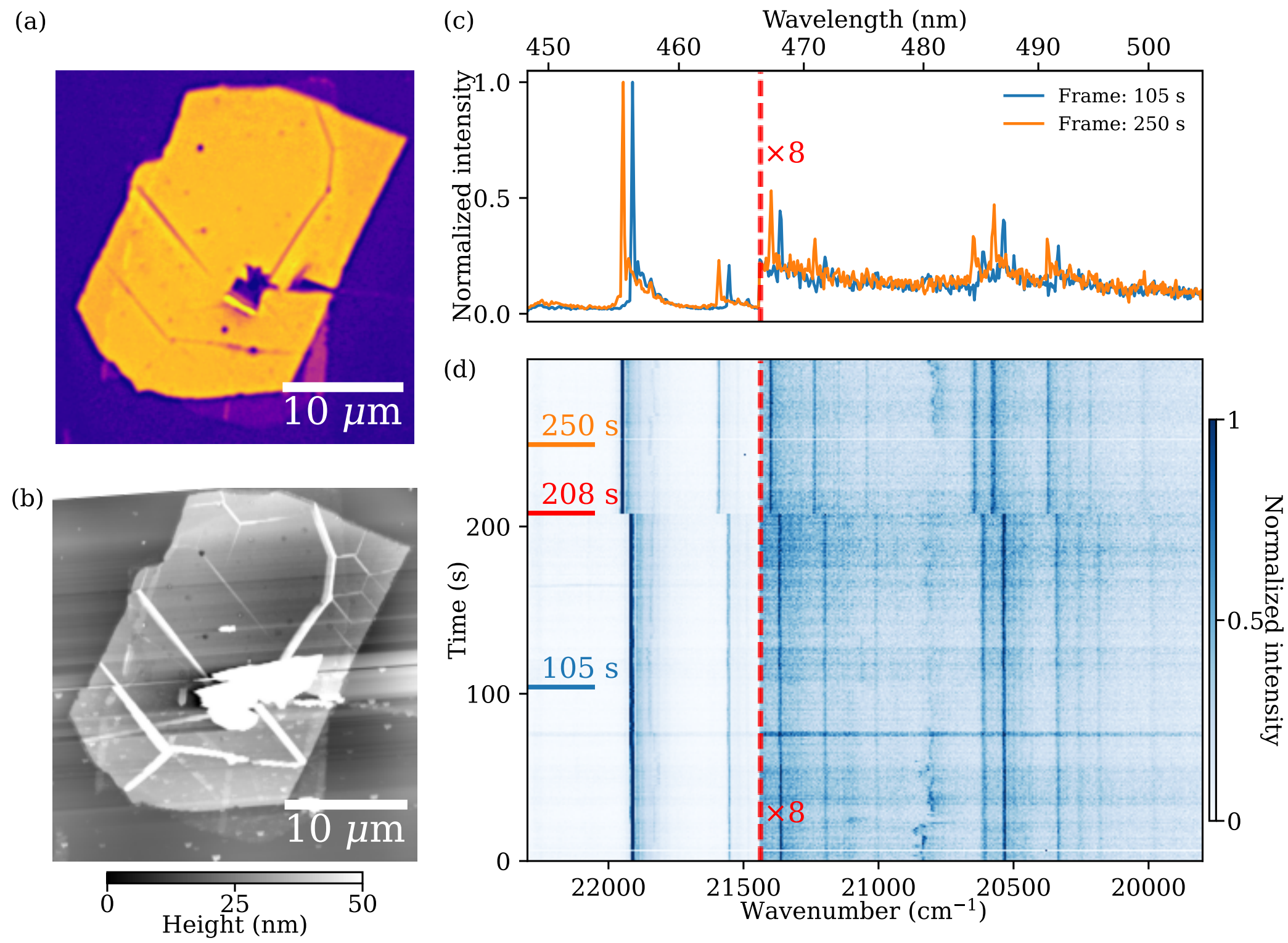

Figure S18: (a), (b) Overview optical microscope and AFM images of annealed sample 2. (c) Representative spectra extracted from the spectral evolution before and after a frequency drift event, corresponding to the horizontal markers in (d). (d) Spectral evolution of one perylene emission site on the annealed sample. The emission remains spectrally stable over several minutes except for a frequency drift at 208 s, marked by the red horizontal line.

7 Hyperspectral imaging

To evaluate the spatial distribution of perylene molecules, we performed hyperspectral imaging to acquire emission spectra over a $10\,\mu m \times 10\,\mu m$ region of interest (ROI). In this measurement, the excitation laser is focused onto a diffraction-limited spot on the sample. The scattered light from the focal point is optically filtered using long-pass filters to block the excitation laser. Then, the remaining fluorescence signal passes through a confocal pinhole

to suppress the background fluorescence and stray light. The signal is subsequently coupled into the spectrometer. After recording the spectrum at one spot, the laser focus is scanned across the entire ROI by using a steering mirror integrated in a 4f-telecentric imaging system, as shown in Fig. **S7**.

A fluorescence intensity map can be generated by integrating the spectral intensity at each scanned position, as shown in Figs. 5(c),(g) of the main manuscript. However, not all the strong fluorescence observed in our samples originates from perylene. For example, broad emission bands at around 570 nm and 620 nm are also detected in our sample [see the left flank of black curves in Figs. **S19**(a–d)]. These two bands match the spectrum of defect-related single photon emitters in hBN .[16,17] Therefore, instead of relying solely on fluorescence intensity, we identify perylene emission sites with spectral features across the hyperspectral map matched to a reference vibronic fingerprint shown in Fig. 2(c) of the manuscript. For the datasets recorded under 450 nm excitation, the identification is performed using the local normalized cross-correlation (LNCC)[18] method, i.e., a sliding-window zero-mean normalized cross-correlation. LNCC matching considers not only the relative spacing between vibronic peaks but also the overall spectral envelope, which improves the matching confidence for spectra with clean perylene fingerprints. By contrast, for the measurements conducted under 423 nm excitation, the matching is only based on the spacing between vibronic peaks. The higher photon energy of 423 nm lasers is more likely to excite additional background fluorescence, possibly from organic residues or other unidentified species in the sample. These background contributions may introduce broad spectral features within the perylene emission window and distort the profile-based LNCC matching result. Thus, peak-spacing matching is used to identify the fingerprints mainly through the relative spacing between dominant emission peaks of perylene, making the analysis less sensitive to variations in the complex spectral background.

7.1 LNCC matching

To do the LNCC matching, the spectra are converted to the wavenumber domain before pattern matching is conducted. Besides, both the reference pattern and the target spectrum are interpolated onto an evenly spaced wavenumber grid.

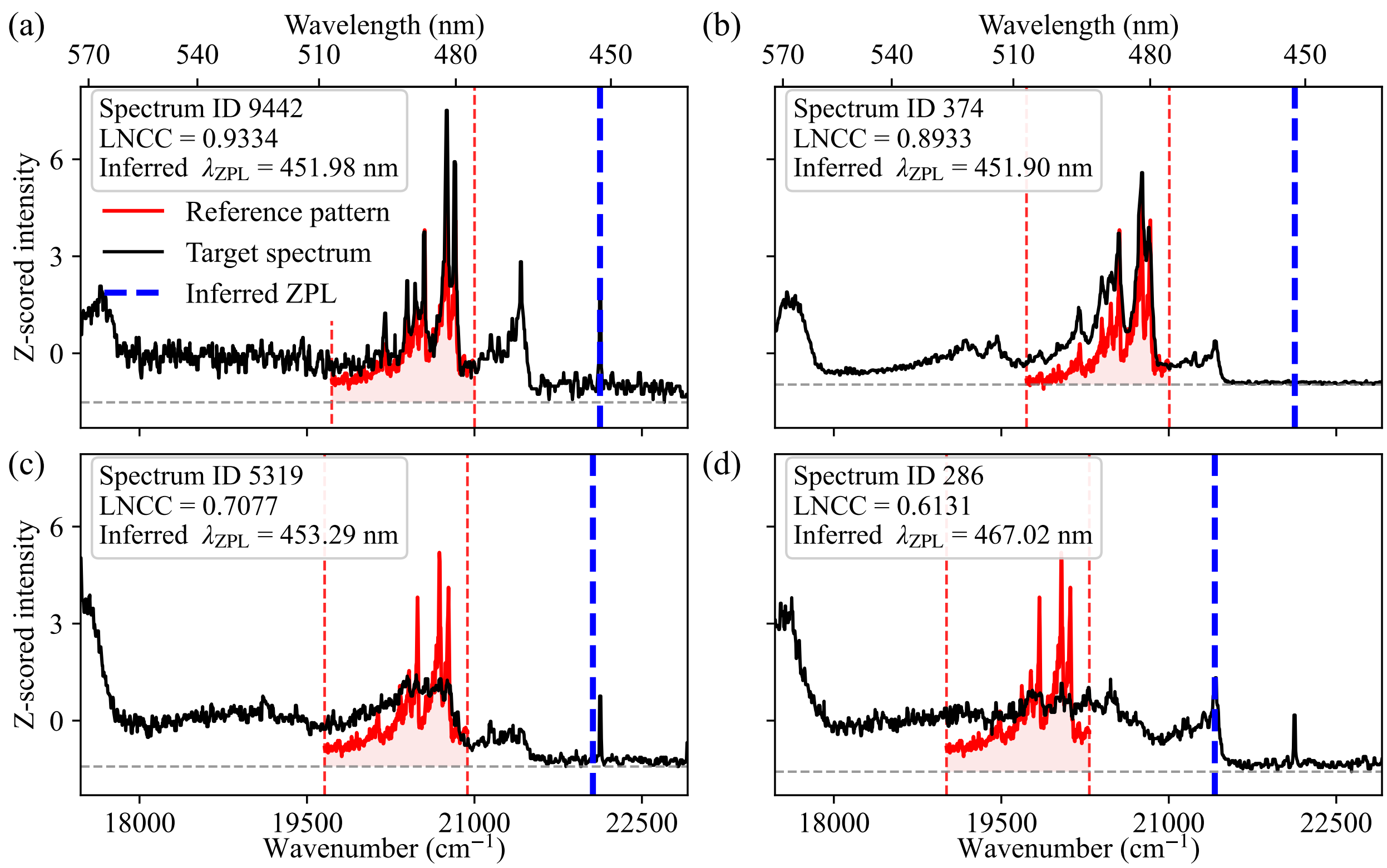

Figure S19: Representative examples of LNCC matching of perylene spectra. In all panels, the black and red curves denote the target spectrum and the reference pattern, respectively, while the blue dashed line marks the inferred ZPL position. (a) A high-confidence case with narrow spectral features and clear correspondence to the reference pattern. (b) A broader spectrum case. The overall lineshape and peak positions remain consistent with the reference, supporting reliable ZPL inference. (c) A case where peak positions are assigned, but the overall profile fails to match; therefore, it is regarded as unreliable. (d) A low-LNCC case, where no clear spectral correspondence can be identified, indicating an invalid match.

The reference template window, shown by the red shaded area in Figs. **S19**(a–d), is denoted by x_i, with $i = 1, 2, \ldots, N$, and the target spectrum is written as y_j, with $j = 1, 2, \ldots, M$, where $M > N$. The reference window then slides across the target spectrum, shown by the black curves in Figs. **S19**(a-d), to evaluate the correlation at different lag po-

sitions. For each lag position k, the corresponding target window is defined as $y_i^{(k)} = y_{k+i-1}$, with $i = 1, 2, \ldots, N$. Here, we apply Z-score normalization to each local target window rather than to the entire target spectrum. The corresponding Z-scores of the reference window and the normalized target window at lag k are written as $Z_x(i) = (x_i - \mu_x)/s_x$, and $Z_y^{(k)}(i) = (y_i^{(k)} - \mu_k)/s_k$, with $i = 1, 2, \ldots, N$, where μ_x and s_x are the mean and standard deviation of the reference window, while μ_k and s_k are the mean and standard deviation of the k–th target window. The correlation coefficient at lag position k is then calculated as

$$\mathrm{LNCC}(k) = \frac{1}{N} \sum_{i=1}^{N} Z_x(i)\, Z_y^{(k)}(i). \tag{7}$$

Compared with a global normalization of the entire spectrum, LNCC suppresses the influence of absolute scale differences and reduces the risk that a few strong coincident features or slow baseline variations dominate the general trend.[19] The maximum LNCC value over all lag positions is taken as the matching score for each spatial position and then used to construct correlation maps shown in Figs. 5(d),(h) of the main manuscript.

Four representative evaluation results using LNCC are shown in Figs. **S19**(a–d). In many cases of the emission spectra, the excitation wavelength lies close to the emitters' ZPL, resulting in the ZPL transition being filtered out by the longpass filter. However, when a reliable vibronic fingerprint match is obtained, the ZPL position can still be reconstructed from the known spectral spacing between the ZPL and vibronic peaks, as shown by the dashed blue lines in Figs. **S19**(a–d).

7.2 Peak-spacing matching

For the datasets measured under 423 nm excitation, perylene emission sites are identified using a peak-spacing-based fingerprint matching method. After the spectra are converted to the wavenumber domain and interpolated onto an evenly spaced wavenumber grid, the characteristic peaks of the reference spectrum are extracted, which are marked with red dots

in Fig. **S20**(a). These peaks are then used to define the relative peak spacing. For each target spectrum, a smoothly varying baseline is estimated from the background fluorescence and subtracted, followed by peak detection based on the local prominence of the baseline-corrected spectra.

Candidate perylene fingerprints are then generated by assigning target peaks as possible characteristic peaks and comparing the expected fingerprint peak positions with the measured data. A candidate is accepted only when a sufficient number of reference fingerprint features are found within the predefined wavenumber tolerance, together with additional constraints on the relative prominence of the dominant peaks. Since multiple perylene fingerprints may coexist within the same spectrum, candidates sharing too many of the same target peaks with others are removed to avoid double counting. The remaining candidates are assigned as possible individual perylene emission sites.

Representative matching results obtained using this peak-spacing method are shown in Figs. **S20**(b-d), where peaks assigned to different perylene molecules are plotted in different colors. Furthermore, corresponding ZPL wavelength of each recognized molecule can also be reliably inferred. Compared with the spectra shown in Fig. **S19**, these spectra recorded under 423 nm excitation may suffer from reduced photon collection efficiency at the detection window from approximately 440 nm to 460 nm, due to chromatic aberration. Nevertheless, multiple perylene molecules can still be clearly recognized from their characteristic fingerprints, even when the overall spectral envelope is distorted.

Then, emission sites with inferred ZPL positions can be spatially mapped across the hyperspectral scan. Spectral matching may still produce isolated false-positive pixels due to noise or accidental partial similarity to the reference pattern. Therefore, the identification of valid emission sites is based not only on spectral matching but also on spatial continuity.

For each spatial position with a valid spectrum, we consider its planar coordinates $\mathbf{r}_i = (x_i, y_i)$ and inferred ZPL wavelength λ_i. A pixel is first retained as a candidate pixel only when it satisfies specific LNCC thresholds for LNCC-based matching or exhibits sufficient

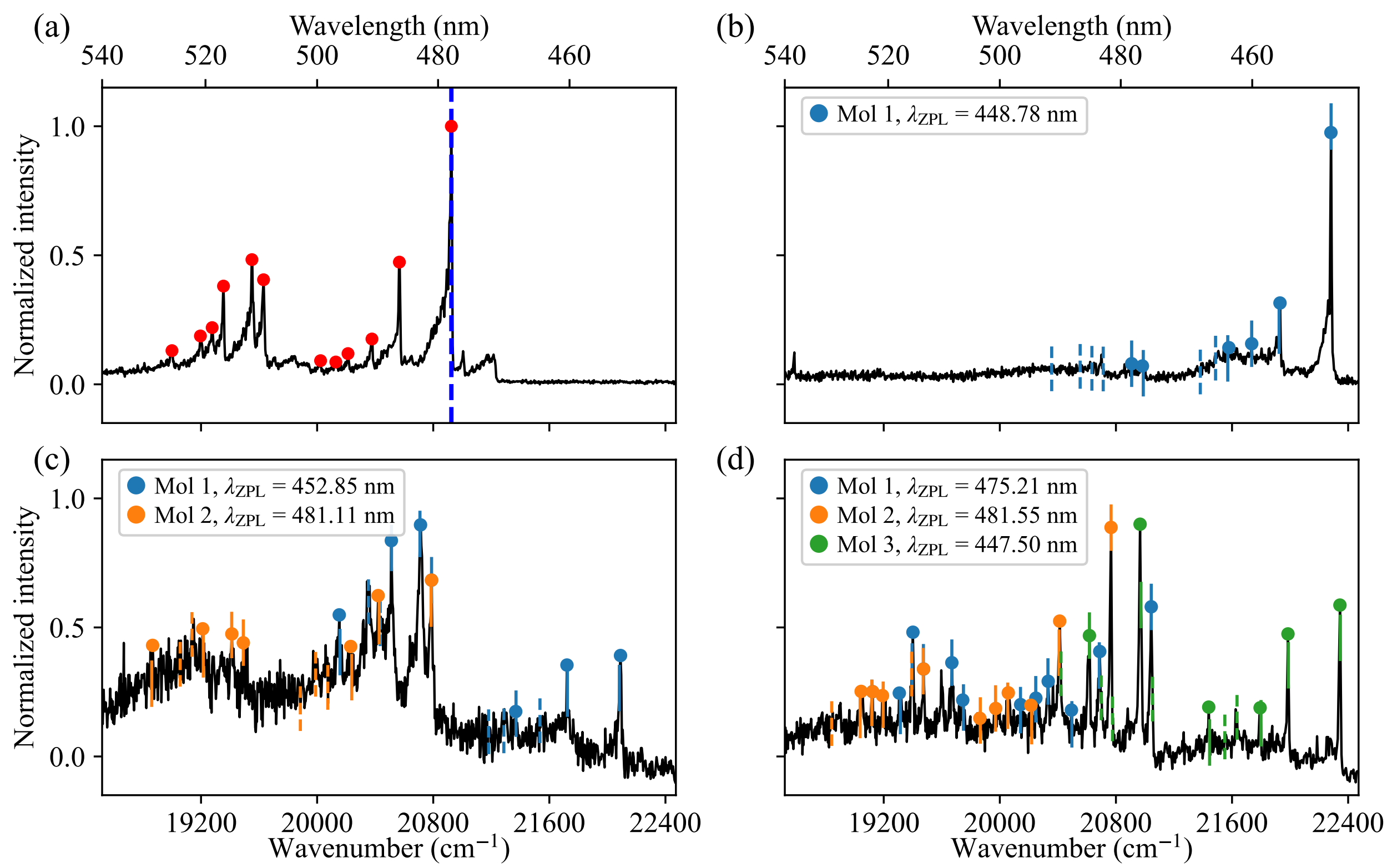

Figure S20: The reference spectrum and representative examples of peak-spacing matching of perylene spectra. In all panels, the black curves denote the measured spectra. (a) Reference perylene spectrum used to define the vibronic fingerprint. The red markers indicate the characteristic peaks used for the peak-spacing analysis, while the blue dashed line marks the ZPL position. (b–d) Representative target spectra analyzed using the peak-spacing method. Colored markers denote peaks assigned to different perylene molecules, and the corresponding inferred ZPL positions are listed in the legends. Dots and solid lines indicate the identified transition peaks, while dashed lines mark the expected peak positions based on the reference pattern.

perylene fingerprint features in the peak-spacing-based matching. The retained candidate pixels are then grouped using a graph-based grouping rule. Two candidate pixels i and j are connected when they satisfy both a spatial proximity condition, $\|\mathbf{r}_i - \mathbf{r}_j\| \leq d_0$, and a spectral consistency condition, $|\lambda_i - \lambda_j| \leq \Delta\lambda_0$, where d_0 is the maximum spatial linking distance and $\Delta\lambda_0$ is the tolerance for ZPL position. The connected components of this graph are then taken as preliminary emission ensembles.

To recover edge pixels that likely belong to the same emission origin, a single-pass extension step is further applied. For a specific ensemble E, a candidate pixel j identified by

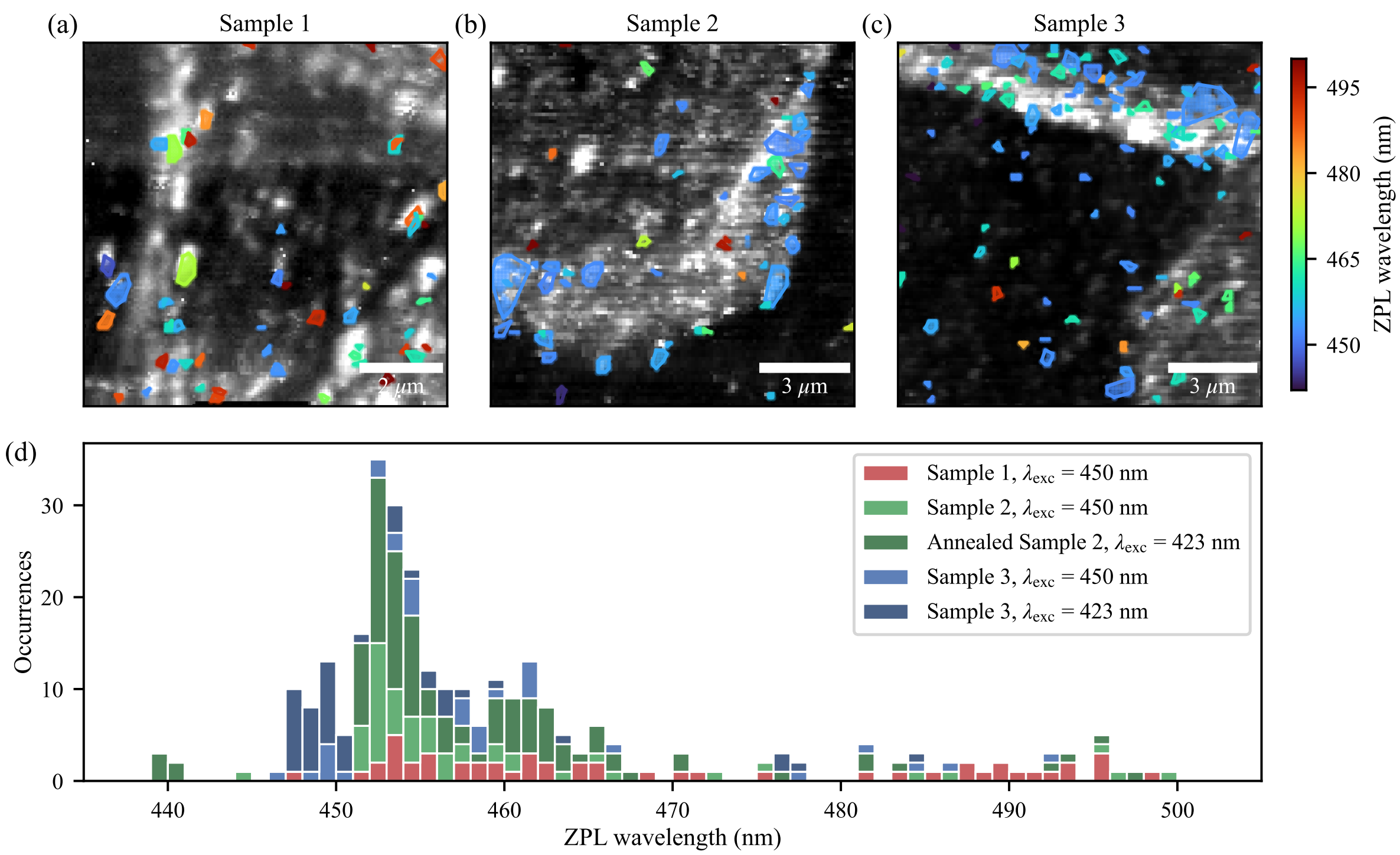

Figure S21: Spatial distribution of emission sites and corresponding histograms of inferred ZPL wavelength across the three samples. (a–c) Overlay maps of emission ensembles on hyperspectral maps of Samples 1–3. The color of the ensembles reflects the inferred ZPL wavelength. (d) Summary histogram of perylene ZPL wavelength across samples and excitation wavelengths.

a KD-tree-based search [20] can be added to ensemble E if it is located within the extension radius and ZPL wavelength tolerance.

After grouping and extension, ensembles containing fewer than three valid candidate pixels are neglected because they are more likely to originate from noise than from stable emission. The retained ensembles are then overlaid on the hyperspectral fluorescence map, with the color indicating their corresponding ZPL wavelengths, together with the overall histogram of the ZPL wavelength throughout the sample shown in Fig. **S21**. The spatial maps reveal substantial differences in the distribution of emission sites. In the pristine stacking region, the emission ensembles are generally smaller and spatially confined. In contrast, in the morphologically irregular region, emission is distributed over extended areas, where multiple sites can exhibit similar spectral features, hinting at similar local environments

within each extended region. In addition, the extracted ZPL wavelengths across all emission ensembles span a broad range from 440 nm to 500 nm, as shown in Fig. **S21**(d), indicating heterogeneous local insertion configurations in the sample.

A comparison of the spectral performance of perylene in different matrices, as well as terrylene adsorbed on an hBN surface, is shown in Tab. **S3**. We observe that the linewidth for our measurement is significantly higher than that for perylene in organic matrices. We attribute this to a possibly higher pure dephasing rate of perylene embedded in hBN. This also would explain why we did not observe power broadening, as dephasing would be the dominant broadening mechanism here.

Table S3: Comparison of perylene (and terrylene at the bottom) as guest single molecule in different host systems. Except for our own measurements of perylene encapsulated in hBN and perylene in anthracene, the information for all the other displayed guest-host systems was taken from Adhikari et al.[21] For perylene in anthracene, there is no single molecule information available.

Guest	Host	Linewidth (MHz)	λ_{ZPL} (nm)
Perylene	encapsulated in hBN	≈ 2800	≈ 440 - 500
Perylene	Polyethylene	≈ 100	≈ 450
Perylene-d12	Dibenzothiophene	≈ 58	≈ 454
Perylene	Anthracene	no single molecule observation	≈ 449
Perylene	Biphenyl	≈ 140	≈ 445
Perylene	n-Nonane	≈ 34	≈ 444
Perylene	o-Dichlorobenzene	≈ 53	≈ 447
Terrylene	on hBN surface	≈ 450	≈ 582

References

(1) Novoselov, K. S.; Geim, A. K.; Morozov, S. V.; Jiang, D.; Zhang, Y.; Dubonos, S. V.; Grigorieva, I. V.; Firsov, A. A. Electric field effect in atomically thin carbon films. *Science* **2004**, *306*, 666–669.

(2) Neese, F. Software Update: The ORCA Program System—Version 6.0. *WIREs Computational Molecular Science* **2025**, *15*, e70019, e70019 CMS-1186.R1.

(3) Lee, C.; Yang, W.; Parr, R. G. Development of the Colle-Salvetti correlation-energy formula into a functional of the electron density. *Phys. Rev. B* **1988**, *37*, 785.

(4) Becke, A. D. Density-functional thermochemistry. III. The role of exact exchange. *The Journal of Chemical Physics* **1993**, *98*, 5648–5652.

(5) Caldeweyher, E.; Ehlert, S.; Hansen, A.; Neugebauer, H.; Spicher, S.; Bannwarth, C.; Grimme, S. A generally applicable atomic-charge dependent London dispersion correction. *The Journal of Chemical Physics* **2019**, *150*, 154122.

(6) Casida, M. E. Time-dependent density functional response theory of molecular systems: theory, computational methods, and functionals. *Theoretical and Computational Chemistry* **1996**, *4*, 391–439.

(7) Hjorth Larsen, A.; Jørgen Mortensen, J.; Blomqvist, J.; Castelli, I. E.; Christensen, R.; Dułak, M.; Friis, J.; Groves, M. N.; Hammer, B.; Hargus, C.; others The atomic simulation environment—a Python library for working with atoms. *Journal of Physics: Condensed Matter* **2017**, *29*, 273002.

(8) Batatia, I. et al. A foundation model for atomistic materials chemistry. *The Journal of Chemical Physics* **2025**, *163*, 184110.

(9) Alkauskas, A.; Buckley, B. B.; Awschalom, D. D.; Van de Walle, C. G. First-principles theory of the luminescence lineshape for the triplet transition in diamond NV centres. *New Journal of Physics* **2014**, *16*, 073026.

(10) Wen, F.; Dong, G. Determining the molecular Huang-Rhys factor via scanning-tunneling- microscopy–induced luminescence. *Phys. Rev. A* **2024**, *109*, 042817.

(11) Grushka, E. Characterization of exponentially modified Gaussian peaks in chromatography. *Analytical Chemistry* **1972**, *44*, 1733–1738.

(12) Parker Jr, J.; Feldman, D.; Ashkin, M. Raman scattering by silicon and germanium. *Physical Review* **1967**, *155*, 712.

(13) Ciancico, C.; Torre, I.; Terrés, B.; Moreno, A.; Smit, R.; Watanabe, K.; Taniguchi, T.; Orrit, M.; Koppens, F.; Reserbat-Plantey, A. Optical detection of charge defects near a graphene transistor using the Stark shift of fluorescent molecules. *APL Quantum* **2025**, *2*, 036101.

(14) Kulzer, F.; Kummer, S.; Matzke, R.; Bräuchle, C.; Basché, T. Single-molecule optical switching of terrylene in p-terphenyl. *Nature* **1997**, *387*, 688–691.

(15) Bordat, P.; Brown, R. Elucidation of optical switching of single guest molecules in terrylene/p-terphenyl mixed crystals. *Chem. Phys. Lett* **2000**, *331*, 439–445.

(16) Xu, Z.-Q. et al. Single photon emission from plasma treated 2D hexagonal boron nitride. *Nanoscale* **2018**, *10*, 7957–7965.

(17) Zeng, L.; Zhang, S.; Meng, J.; Chen, J.; Jiang, J.; Shi, Y.; Huang, J.; Yin, Z.; Wu, J.; Zhang, X. Single-Photon Emission from Point Defects in Hexagonal Boron Nitride Induced by Plasma Treatment. *ACS Applied Materials & Interfaces* **2024**, *16*, 24899–24907.

(18) Lewis, J. P. Fast Template Matching. Vision Interface. Quebec City, Canada, 1995; pp 120–123.

(19) Avants, B. B.; Epstein, C. L.; Grossman, M.; Gee, J. C. Symmetric diffeomorphic image registration with cross-correlation: evaluating automated labeling of elderly and neurodegenerative brain. *Medical Image Analysis* **2008**, *12*, 26–41.

(20) Friedman, J. H.; Bentley, J. L.; Finkel, R. A. An algorithm for finding best matches in logarithmic expected time. *ACM Transactions on Mathematical Software (TOMS)* **1977**, *3*, 209–226.

(21) Adhikari, S.; Smit, R.; Orrit, M. Future Paths in Cryogenic Single-Molecule Fluorescence Spectroscopy. *The Journal of Physical Chemistry C* **2024**, *128*, 3–18.
